# Citizen science and online data: Opportunities and challenges for snake ecology and action against snakebite

**DOI:** 10.1016/j.toxcx.2021.100071

**Published:** 2021-06-22

**Authors:** Andrew M. Durso, Rafael Ruiz de Castañeda, Camille Montalcini, M. Rosa Mondardini, Jose L. Fernandez-Marques, François Grey, Martin M. Müller, Peter Uetz, Benjamin M. Marshall, Russell J. Gray, Christopher E. Smith, Donald Becker, Michael Pingleton, Jose Louies, Arthur D. Abegg, Jeannot Akuboy, Gabriel Alcoba, Jennifer C. Daltry, Omar M. Entiauspe-Neto, Paul Freed, Marco Antonio de Freitas, Xavier Glaudas, Song Huang, Tianqi Huang, Yatin Kalki, Yosuke Kojima, Anne Laudisoit, Kul Prasad Limbu, José G. Martínez-Fonseca, Konrad Mebert, Mark-Oliver Rödel, Sara Ruane, Manuel Ruedi, Andreas Schmitz, Sarah A. Tatum, Frank Tillack, Avinash Visvanathan, Wolfgang Wüster, Isabelle Bolon

**Affiliations:** a Department of Biological Sciences, Florida Gulf Coast University, Ft. Myers, FL, USA; b Institute of Global Health, Department of Community Health and Medicine, Faculty of Medicine, University of Geneva, Geneva, Switzerland; c World Health Organization, Geneva, Switzerland; d University of Bern, Bern, Switzerland; e Citizen Science Center Zürich (ETH Zürich and University of Zürich), Zürich, Switzerland; f University of Geneva, Geneva, Switzerland; g École polytechnique fédérale de Lausanne, Geneva, Switzerland; h The Reptile Database, Richmond, VA, USA; i Virginia Commonwealth University, Richmond, VA, USA; j Suranaree University of Technology, Nakhon Ratchasima, Thailand; k R. J. Gray Ecology, New Smyrna Beach, FL, USA; l HerpMapper, St. Paul, MN, USA; m HerpMapper, Cedar Rapids, IA, USA; n HerpMapper, Champaign, IL, USA; o Indian Snakes, Kottayam, Kerala, India; p Instituto Butantan, São Paulo, São Paulo, Brazil; q University of São Paulo, São Paulo, São Paulo, Brazil; r University of Kisangani, Kisangani, Democratic Republic of the Congo; s University of Geneva Hospitals, Geneva, Switzerland; t Flora & Fauna International, Cambridge, England, UK; u Global Wildlife Conservation, Austin, TX, USA; v Universidade Federal do Rio Grande, Rio Grande, Rio Grande do Sul, Brazil; w Reptile Database, Scotts Mills, OR, USA; x Murici Ecological Station, Murici, Alagoas, Brazil; y University of the Witwatersrand, Johannesburg, South Africa; z Bangor University, Bangor, Wales, UK; aa Anhui Normal University, Wuhu, Anhui, China; ab Rutgers University, New Brunswick, NJ, USA; ac Madras Crocodile Bank Trust, Mahabalipuram, Tamil Nadu, India; ad Toho University, Funabashi, Japan; ae EcoHealth Alliance, New York, NY, USA; af Tribhuvan University, Biratnagar, Nepal; ag Northern Arizona University, Flagstaff, AZ, USA; ah Global Biology, Birr, Switzerland; ai Institute of Development, Ecology, Conservation & Cooperation, Rome, Italy; aj Museum für Naturkunde - Leibniz Institute for Evolution and Biodiversity Science, Berlin, Germany; ak Rutgers University, Newark, NJ, USA; al Museum d'Histoire naturelle Geneve, Geneva, Switzerland; am University of North Georgia, Dahlonega, GA, USA; an Friends of Snakes Society, Hyderabad, Telangana, India; ao Molecular Ecology and Fisheries Genetics Laboratory, School of Natural Sciences, Bangor University, Bangor, Wales, UK

**Keywords:** Snakes, Biodiversity, Photography, Snakebite, Endemism, Online data, Citizen science, Data science

## Abstract

The secretive behavior and life history of snakes makes studying their biology, distribution, and the epidemiology of venomous snakebite challenging. One of the most useful, most versatile, and easiest to collect types of biological data are photographs, particularly those that are connected with geographic location and date-time metadata. Photos verify occurrence records, provide data on phenotypes and ecology, and are often used to illustrate new species descriptions, field guides and identification keys, as well as in training humans and computer vision algorithms to identify snakes. We scoured eleven online and two offline sources of snake photos in an attempt to collect as many photos of as many snake species as possible, and attempt to explain some of the inter-species variation in photograph quantity among global regions and taxonomic groups, and with regard to medical importance, human population density, and range size. We collected a total of 725,565 photos—between 1 and 48,696 photos of 3098 of the world's 3879 snake species (79.9%), leaving 781 “most wanted” species with no photos (20.1% of all currently-described species as of the December 2020 release of The Reptile Database). We provide a list of most wanted species sortable by family, continent, authority, and medical importance, and encourage snake photographers worldwide to submit photos and associated metadata, particularly of “missing” species, to the most permanent and useful online archives: The Reptile Database, iNaturalist, and HerpMapper.

## Highlights

• We collected 725,565 photos of 3098 snake species (79.9%), leaving 781 “most wanted” species with no photos.
• A total of 107 countries, mostly developed, small, or insular, have all species represented.
• Medically-important venomous snakes have more photos in online databases than other species.
• We provide a list of most wanted species sortable by family, continent, authority, and medical importance.
• We encourage photographers to submit photos and metadata to The Reptile Database, iNaturalist, & HerpMapper.

## Graphical abstract

**Figure:**
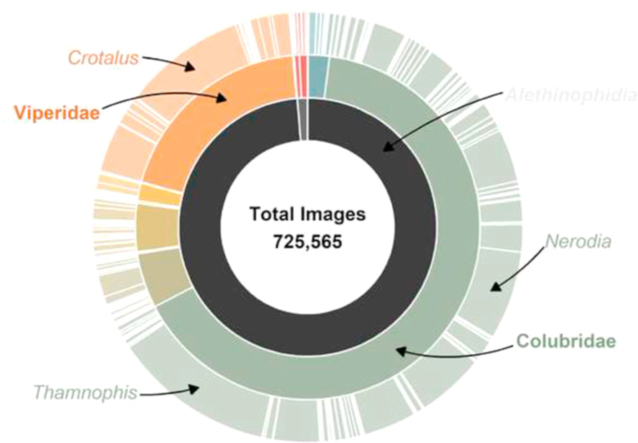

## 1 Introduction

Our understanding of the global diversity and distribution of the nearly 3900 species of snakes remains incomplete (Böhm et al., 2013; Roll et al., 2017). This situation is partially attributable to the widespread fear of snakes (Tierney and Connolly, 2013), even among scientists and academics, but largely to the secretive behavior and life history of snakes (Steen, 2010), which limits sample sizes in ecological studies (Bonnet et al., 2002; Seigel, 1993) and hinders the assembly of country-level species checklists in some parts of the world (e.g., Bauer et al., 2017; Branch et al., 2019; Marques et al., 2018). Range maps for most snake species recently became available (Roll et al., 2017), although changes in taxonomy and the rapid rate of new species description create challenges for scientists wishing to use such resources. The Reptile Database (Uetz et al., 2021) provides regular updates on snake taxonomy, but the complexity of biological nomenclature and the absence of an automated interface with other online data portals, such as VertNet and Genbank, makes connecting names among sources challenging. Molecular systematics has produced an increase in species description rates (Simkins et al., 2020), and the number of new snake species described in the 21st century so far has averaged 33 per year (Uetz et al., 2021).

Although preserved specimens remain the gold standard in biological research in terms of permanence and reproducibility (Ceríaco et al., 2016), one of the most useful, most versatile, and easiest to collect types of biological data are photographs (Borges et al., 2020; Leipzig et al., 2021). Particularly when combined with geographic location and date-time information, photos of snakes and other wild animals have a variety of potential applications, including serving as a verifiable basis for mapping occurrence records, as data sources to answer ecological questions and to map and quantify gradients of phenotypic variation, as reference material for new species descriptions, for use in illustrating field guides and identification keys, as training material for improving identification skills, as training and testing material for computer vision algorithms, and as reference material for identifying snakes in snakebite cases.

The development of citizen science platforms that emphasize the collection and classification of biological and ecological data (Bonney et al., 2009; Dickinson et al., 2010; Kullenberg and Kasperowski, 2016), including photos, has the potential to reduce this shortfall (Chandler et al., 2017; Goiran and Shine, 2019; Troia and McManamay, 2016). Two major citizen science platforms contribute the vast majority of the publicly-available structured online data on snake distribution and appearance: iNaturalist, which includes snakes as well as all other taxa, and HerpMapper, which is specific to amphibians and reptiles. Both iNaturalist and HerpMapper require users to enter structured data (date, time, location, and species). HerpMapper requires a voucher photo, and iNaturalist strongly encourages voucher photos. Other online sources of snake photos exist, but many lack structured data. For example, CalPhotos and Wikimedia collect photos but do not require date, time, and location information to be associated with them. The photo sharing website Flickr, home to a community of photographers with a serious interest in biodiversity, allows but does not require users to include this information, and the social media sites Twitter and Facebook contain millions of such photos but little to no structured data. Finally, many private collections of snake photos exist, most of which are challenging to access. Marshall et al. (2020a) recently published an inventory of online reptile images, conducted concurrently with our study, which we incorporate here and on which we build by adding 313,668 photos and 281 species of snakes.

**Fig. 1:**
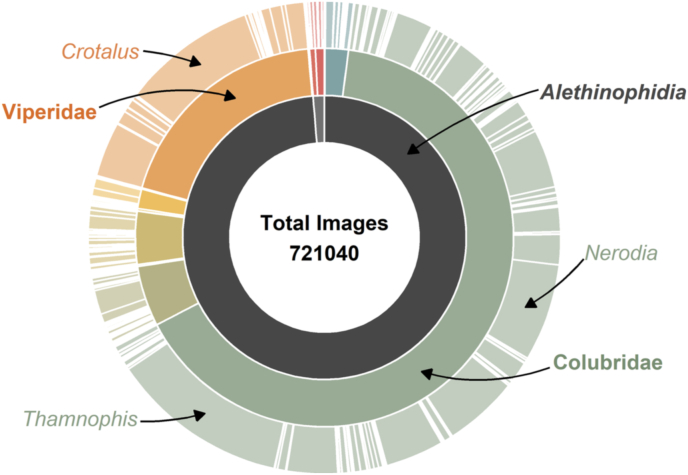
Taxonomic structure of dataset. The innermost ring represents infraorders: blindsnakes (Scolecophidia; light gray) and all other snakes (Alethinophidia; dark gray). The middle ring represents families, and the outermost ring represents genera. Width of slices is scaled to the number of photos.

We attempted to combine these resources to assemble the largest set of snake photos to date, which contains at least one photo of 3098 (79.9%) of all 3879 valid species as of the December 2020 release of The Reptile Database (Fig. 1). Controlling for variation among global regions and between scolecophidian blindsnakes (ca. 450 ecologically-similar fossorial non-venomous species that are generally smaller and even more cryptic than other snake species but which might not be monophyletic; Miralles et al., 2018) and alethinophidian (all other) snakes, we asked several questions about what explains the variation among species in the number of photos, namely:

• Do species with larger geographic ranges have more photos?
• Do species with a higher human population density in their range have more photos?
• Do species described earlier have more photos?
• Do medically-important venomous snakes have more photos than non-venomous species?

## 2 Methods

### 2.1 Photo sources

We collected photo URLs, species names, and other metadata where available (detailed below) from Internet sources. Here we describe each source and our method for collecting and cleaning, where necessary, the above information. Our target was color photographs taken of wild living snakes, one individual snake per photo, as many photos as possible per species. We did not consider photo quality (i.e., composition, resolution, visibility of the entire snake or of diagnostic features) to be of particular importance, because we were interested in representing the kinds of photos that would be taken by a range of photographers, from expert to amateur.

1. **iNaturalist** is a prominent citizen science platform where users submit photos or other media of any organism from any location around the world. The user community of ~1.5 million contributes crowd-sourced identification conforming to curated taxonomic names, of which The Reptile Database (Uetz et al., 2021) is the single most comprehensive and up-to-date online provider for reptiles. We used the iNaturalist Data Export Tool (https://www.inaturalist.org/observations/export) with the query string quality_grade = research&identifications = any&captive = false&taxon_id = 85553 corresponding to Research Grade observations (i.e., dated and georeferenced observations, with at least two users identifying the species and the majority of all identifications are in agreement), not in captivity, and in the taxonomic clade Serpentes. The date range included all observations that had achieved Research Grade as of 5 February 2021. The curated taxonomic names in iNaturalist follow The Reptile Database, with relatively few exceptions that we fixed manually. It was not apparent at first that the iNaturalist Data Export Tool counts each observation only once, even if observations have >1 photo (see GBIF).
2. **The Global Biodiversity Information Facility (GBIF)** aggregates biodiversity data from hundreds of institutions across the world. We queried GBIF on 6 February 2021 for all ‘human observation’ records of snakes, using the list of family names in Appendix 1. The vast majority (404,793; 83.5%) of 484,563 records with media returned were iNaturalist records, leaving 79,770 photos from 45 other data contributors. Most of these are images of preserved specimens in natural history museums, with a handful of computerized tomography (CT) scans, sound recordings, or other miscellaneous images related to snakes, which were not useful to us. However, GBIF provides two important advantages over direct exports from iNaturalist: 1) the data downloads are archived with DOIs (GBIF.org, 2021) and 2) iNaturalist observations with more than one photograph are counted. The GBIF platform only incorporates photos with CC0, CC-BY and CC-BY-NC licenses, whereas the default users setting in iNaturalist is All Rights Reserved (ARR). Because we wanted to understand the full scope of online snake photos, we used the higher count of either method, but spot-checking revealed that even doing so resulted in an undercount, because there are many ARR observations with >1 photo. The average ± S.D. number of photos per iNaturalist snake record in GBIF is 1.6 ± 1.2 (max = 71, but 93.8% of iNaturalist records have 3 or fewer photos); thus, we expect that iNaturalist contains several thousand additional snake photos that are not indexed in GBIF and are not easily quantified using iNaturalist's native tools.
3. **HerpMapper** is the largest global reptile and amphibian citizen science platform (O'Donnell and Durso, 2014). Users submit photos or other media of any reptile and amphibian species from any location around the world. There is no identification validation, but the HerpMapper data contributor community is made up of professional herpetologists and amateur herpetological enthusiasts (“herpers”) with a high level of expertise identifying species of snakes and other reptiles. An important feature is that other users of HerpMapper and the general public do not have access to exact locality data; only verified HerpMapper Partners have access, and very sensitive records can hidden from public view entirely, in order to protect sensitive locality information. We submitted a data request and received a list of all snake photo URLs on 3 December 2019, which we updated from https://www.herpmapper.org/records?taxon=Serpentes with the help of the HerpMapper admins following Marshall et al. (2020a). The date range now includes all observations that had been submitted as of 5 February 2021. The curated taxonomic names in HerpMapper follow The Reptile Database, with relatively few exceptions that we fixed manually.
4. **CalPhotos** is a University of California Berkeley Natural History Museum photo database, and one of the oldest online image databases specializing in natural history, dating to 1995. The database contains digital images of plants, animals, and other natural history subjects, along with descriptive information including scientific names, provided by the person or organization that contributed the photos. Experts review identification of the photos. We used the Custom Query form at https://calphotos.berkeley.edu/cgi/img_query to collect lists of photos of snakes at the family level, following the format ?where-family = Colubridae. For a list of snake family names and synonyms used, see Appendix 1. By using the options text_only = 1&max = 3000 we generated a single table per family which we then copied into a spreadsheet and summarized in R. The taxonomy does not necessarily follow any one standard (but uses the Reptile Database as source for species names); deviations from The Reptile Database taxonomy were fixed manually. This methodology differs from that used by Marshall et al. (2020a), who used counts of one for all species with photos in CalPhotos.
5. **Wikimedia** Commons is a collection of freely useable media files without a specific biodiversity focus, but which includes many photos of animals. We used the exact process implemented by Marshall et al. (2020a) to query all Reptile Database snake species names and synonyms, discarding page results that did not include the “Articles with ‘species’ microformats” category (for more detail, see http://microformats.org/wiki/species). Because we enforced The Reptile Database taxonomy in our search terms, no correction of taxonomic names was necessary, but we undoubtedly missed a small number of photos that are tagged using e.g., misspelled scientific names or common names only in Wikimedia.
6. **The FitzPatrick Institute of African Ornithology Virtual Museum (VMUS)** hosts a Reptile Atlas of Africa that collects georeferenced photos of largely southern African reptiles. We queried the VMUS for each snake family at the following URL: http://vmus.adu.org.za/vm_search.php?database=sarca&prj_acronym=ReptileMAP&db=sarca&URL=http://sarca.adu.org.za&Logo=images/reptilemap_logo.png&Headline=Reptile%20Atlas%20of%20Africa&Use_main_filter=0&User_id=&Full_name=&serve_sp_list=1&drop_down_list=Latin%20names&assessment=0#familyThe taxonomy does not necessarily follow any one standard; nevertheless, because most contributors are experts, there were sufficiently few deviations from The Reptile Database taxonomy that we fixed them manually.
7. **The Indian Snakebite Initiative (ISI)** Big 4 Mapping Project (http://snakebiteinitiative.in/snake/) is a network of snake rescuers in India who collect photos and location information when they are called to remove a snake from a person's home. About 800 volunteer participants use an Android app to upload photos and metadata. For more detail see http://snakebiteinitiative.in/#/Big4compo. This project collects photos of only the four most medically-important species throughout India (*Bungarus caeruleus*, *Daboia russelii, Echis carinatus*, *Naja naja*). Photos were transferred over Microsoft OneDrive and associated metadata were provided via a one-time admin access to the backend provided by a private arrangement on 19^th^ January 2019.
8. **The Reptile Database** itself curates a collection of reptile photos, largely gathered from private photographers and described by Marshall et al. (2020a). Unlike the others, this data source contains about 700 images of preserved specimens from museum collections, many of which are also represented by live photos.
9. **Flickr** is a photo-sharing website with a sub-community of photographers with a serious interest in biodiversity. Unlike all of the above, there is no site-wide biodiversity focus or structured way for users to tag photos as particular species within a hierarchical taxonomic framework. Marshall et al. (2020a) queried Flickr using their API (28 June 2020) but this took place after Flickr changed from a free platform with unlimited storage to a service that offered a small amount of free image hosting but required paid subscriptions for users with more than 1000 photos in 2019. We collected Flickr data in November 2018 using a python script (https://github.com/cam4ani/snakes/blob/master/get_flickr_data.ipynb), prior to the point when many users may have deleted their accounts or moved them to other platforms. We used all then-valid Reptile Database snake genus names as search terms, as well as the list of families in Appendix 1, and common names in multiple languages taken from Wikipedia page titles (see script for details). We discarded certain genera that have multiple other meanings and would likely have resulted in large numbers of irrelevant photos (e.g., *Arizona*, *Virginia*, *Python*). We did not attempt to remove photos of captive snakes.
10. **Twitter** is not organized into snake or biodiversity-specific groups (although certain hashtags such as #NotACopperhead would lead users to photos of snakes) but represents a rich source of photos that is relatively easy to query. We queried the Twitter public streaming API from 15^th^ May 2019 to 23^rd^ August 2019. Because we were limited to 400 search terms, we identified parts of genus names that corresponded to particular strings that would be uncommon in everyday words (e.g., “ophis”, which is the ending of 21% (111 of 517) of then-valid snake genera; see Appendix 2 for full list) and added to these a list of common name parts (e.g., “viper”, “cobra”; see Appendix 2 for full list). The text of the Tweet, the expanded and display URLs for links and media, text for hashtags, and username were checked for matches. We removed tweets that Twitter had marked as containing sensitive media. We obtained a total of 194,545 images. One of us (AMD) went through a subset (N = 6684) of these images in a private project on Crowdbreaks (Müller and Salathé, 2019) to tag inappropriate, irrelevant, and redundant images; these categories were used to train an image classifier which predicted 16,190 (8.3%) of the total to be relevant. The classifier was based on a ResNet-50 model (He et al., 2016) pre-trained on ImageNet (Deng et al., 2009). The classifier was used to filter images which were predicted to be within the “snake, serpent, ophidian” category and were manually verified to be relevant by AMD. We removed exact image duplicates, leaving us with 7808 images (4% of the original). We then crowd-sourced species-level identifications in the same manner as Durso et al. (2021a)—for each image, participants had to select a species, genus, or family-level taxonomic name matching the then-current Reptile Database taxonomy. Participants could also tag images as “not a snake/contains multiple species” (262 images). We awarded prizes to participants who tagged the most images between 18th November and 28^th^ November 2019 (see https://snakes.citizenscience.ch/en/ranking/autumn-2019). We collected a total of 70,507 image tags, 54,873 of which were at the species level (range 4–33 tags per image, mean ± S.D. = 9.5 ± 3.3). We selected the subset of tagged images that had a single most common species-level tag, had been tagged by at least 3 participants, and where at least 50% of the tags were the same (5019 images). We found images of 323 species (range 1–418 images per species), three of which were not represented in any other data source (1 image each; but see discussion).
11. **Facebook** is home to numerous groups and pages that focus on providing rapid identification of snake photos (Smith et al., 2019), the largest of which includes hundreds of thousands of users and helps to identify hundreds of snake photos per day. Durso et al. (2021a) provided a list of such groups as of 2020. These groups have photo collections of high value. Unfortunately, Facebook no longer supports systematic access via an API that is independent of the user access point, which means that searches are likely non-reproducible and influenced by user language, location, and group access. Marshall et al. (2020a) and others have lamented the same problem, which is especially acute because Facebook likely represents the single largest and most carefully curated online source of snake photos on the web (it is difficult to assess whether Facebook or iNaturalist has more photos because of the limitations described here—Facebook has almost 2000 times as many users but only a small percentage are involved in snake identification/biodiversity sub-communities). In contrast to Twitter, most of the non-snake, viral, duplicate, or otherwise irrelevant images have already been filtered out by diligent moderators and administrators of snake ID groups, and every photo contains a species common and/or scientific name (non-standardized taxonomy) in the caption or comment thread. We attempted but were ultimately unable to access these photos and metadata in a systematic way, but mention them here because of their promise for future use in biodiversity imagery collection.
12. We compiled a list of **professional herpetologists** who we thought might have extensive private snake photo collections. A total of 36 individuals generously provided between 1 and 2132 photos, which were transferred by email or third-party server. About twice this many individuals were contacted but either declined to provide photos or expressed willingness to do so, but never did. Some unknown percentage of these photos may be redundant with other data sources, for instance if these individuals are also contributors to The Reptile Database, iNaturalist, HerpMapper, or CalPhotos. It is important to recognize that all photographers retain ownership and copyright to their own photos.
13. Once we had assembled a list of all species with 0 photos after taking all of the above into account (947 species), we sorted this list by year of species description and examined the **peer-reviewed literature** containing the original species description, when available, for all species described between 1990 and 2020. We reasoned that prior to 1990, it was rare to publish color photos in journals. If the original species description contained one or more color photos of the species in life, we manually extracted these and added them to the dataset. We also extracted photos of other snake species of which we already had photos, if the papers contained any. In this way we were able to add 338 photos of 163 additional species, between one and ten photos per species.

Some data sources (iNaturalist, Flickr) allow filtering by Creative Commons license, whereas the legal ownership of photos on others (Twitter, Facebook) is unclear. We operate under the assumption that all photographers retain copyright to their own photos, regardless of the data source, pursuant to our Data Management Plan on file with the Swiss National Science Foundation (SNSF). In cases where an All Rights Reserved license applied, we simply counted the photo as existing in the data source.

### 2.2 Other data

**Fig. S1:**
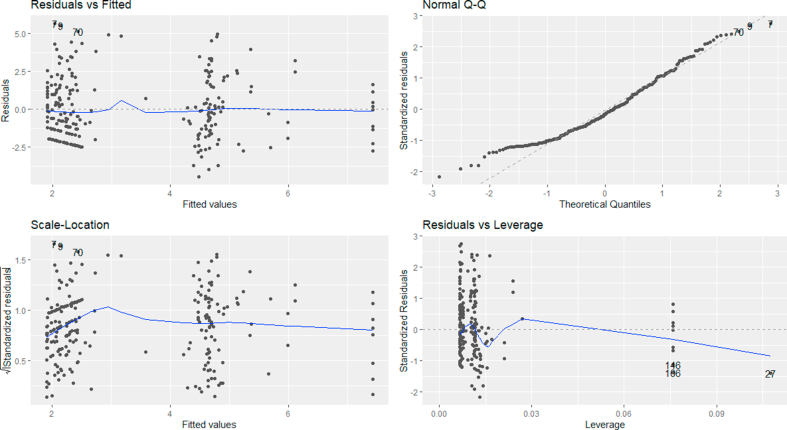
Tests of model assumptions for ln(numberofphotos+1)~ln(yearsafter1758+1)+presenceofnameinGARD(y/n).

We collected range size information in km^2^ from the Global Assessment of Reptile Distributions (GARD) Initiative (http://www.gardinitiative.org/data.html) (Roll et al., 2017). These maps do not include marine snakes (N = 71), species described since 2017 (N = 246), species whose geographic distribution is unknown (N = 11) and a small number of species that are not mapped for no obvious reason (N = 24), so these species were omitted from the spatial analysis. We also updated the names of 171 species where the genus had changed and corrected the names of 52 species where spelling errors or changes to the gender of the specific epithet caused an imperfect match to the December 2020 Reptile Database taxonomy. Where two or more mapped species had been synonymized since 2017, we combined their range maps (14 species combined into 5, affecting 285 photos; see Appendix 3 for complete list). A number of currently-valid species of snakes have been split from mapped species since 2017. When a mapped species had been split into two or more species since the GARD maps were produced, we combined photo counts back to the more widespread species for which we had a map (252 species re-combined into 101, affecting 20% [145,814] of the photos in the dataset; see Appendix 3 for complete list). We used a linear model with the form:

ln(numberofphotos+1)~ln(yearsafter1758+1)+presenceofnameinGARD(y/n)

to ask whether the more widespread *sensu lato* species names in GARD or the more restricted *sensu stricto* species name(s) that have been revalidated since the GARD mapping project had more photos (assumptions tested in Fig. S1). Adding one is to avoid the undefined natural log of 0.

**Fig. 2:**
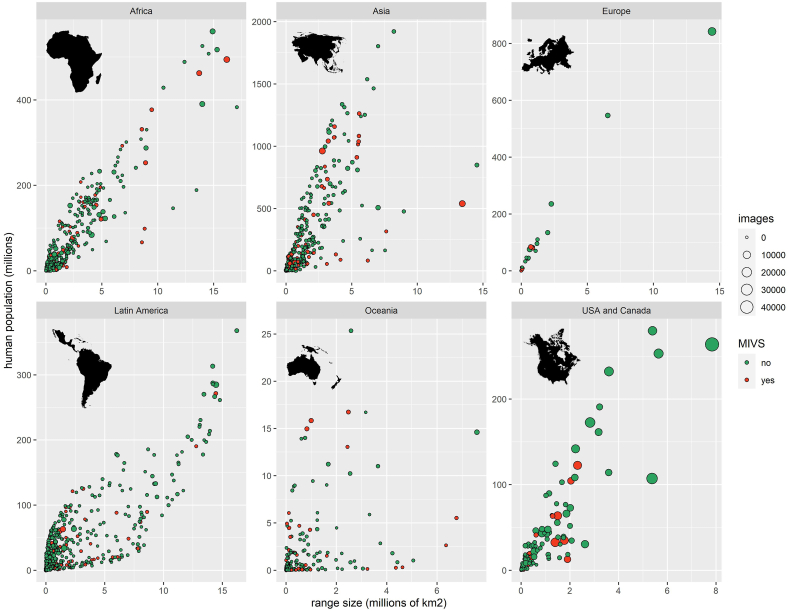
Relationship between range size (millions of km^2^) and human population in range (millions of people) by global region, with point size scaled to number of photos and medically-important venomous snakes (MIVS) in red. Each point is one species. (For interpretation of the references to color in this figure legend, the reader is referred to the Web version of this article.)

We obtained gridded data on human population density (30 arc-second grid cells) from the Center for International Earth Science Information Network (CIESIN) at Columbia University and calculated the mean human population density within each GARD range map polygon using the Zonal Statistics tool of the Spatial Analyst toolbox of ArcGIS v.10.2. We assigned species to global regions using the coords2continent function in the rworldmap package (South, 2011) on the centroid of each GARD range, and creating a custom dictionary for any species that were unmatched because they were not mapped in GARD or their centroids lay outside of a continent. Although we include a marine region in some summaries, this region was not part of our statistical model because range size and population density data are lacking. We examined the relationship between range size and number of people living within the range by global region (Fig. 2).

We used the World Health Organization ((WHO 2016)) list of medically-important venomous snake (MIVS) species as a starting point and updated this list to reflect taxonomic changes and newly described species from medically-important genera since 2016. This resulted in 3313 species with non-medically-important designations and 566 species with medically-important designations. Note that totals may differ below (i.e., 531 MIVS and 2883 non-MIVS mapped by GARD, 499 MIVS and 2859 non-MIVS with complete panel data for analysis) because of the necessity of connecting these names with those from GARD and other sources (see above). This is not intended to be a systematic review of clinical importance; we simply assumed that if a species belonged to a genus that was made up of medically-important species, or a genus not listed by the WHO that had recently been split from another medically-important genus, then it was likely to be of medical importance, although not all potentially life-threatening species are responsible for significant mortality and morbidity in a given area (Silva, 2013) and species that are found in close proximity to humans vary in their contribution to the burden of snakebite (Glaudas, 2021; Udyawer et al., 2021).

The Reptile Database provided data on dates of original description (Uetz, 2010), synonyms, and higher taxonomy, as well as scans of hard-to-find original descriptions. Although we attempted to connect names from the different data sources to those in the Reptile Database using the R package taxize (Chamberlain et al., 2019), we found that a substantial number remained unmatched and had to be connected using custom dictionaries created manually. This issue also affects other biodiversity databases, such as NCBI GenBank (Garg et al., 2019).

### 2.3 Data analysis

**Fig. S2:**
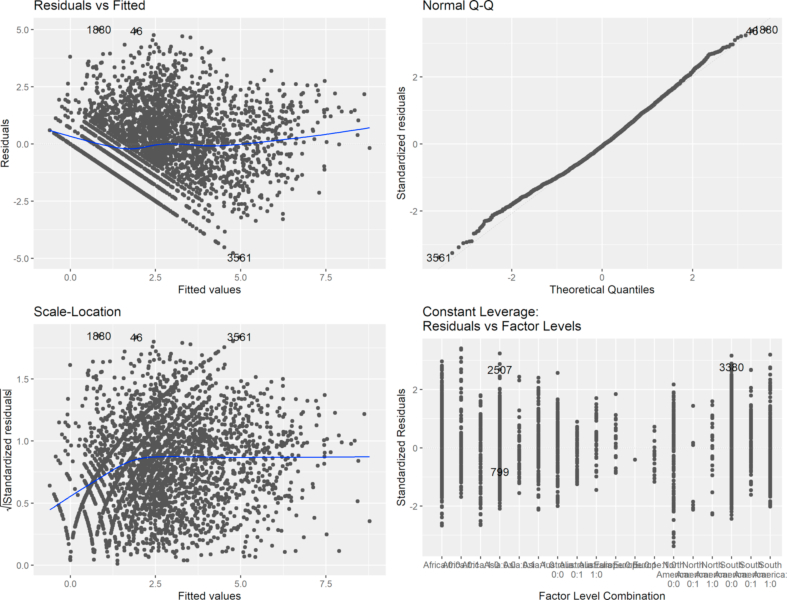
Tests of model assumptions for $ln\left(number\;of\;images+1\right)\;\sim \;\text{ln}\left(range\;size\right)+\text{ln}\left(population\;density\right)+\text{ln}\left(population\;density^{2}\right)+continent+mivs+blindsnake+year$.

We used dredge function in the multi-model inference (MuMIn; Bartón, 2020) package to perform model selection on all subsets of a global linear regression (function lm) model in R 3.6.3 (R Core Team, 2020). We ranked models using AIC_c_. We log-transformed the response variable (number of photos) to meet assumptions of normality and homogeneity of variance of the residuals (Fig. S2), and we log-transformed the continuous explanatory variables to make interpretation of effect sizes more intuitive. Fixed explanatory variables for each species were:

• geographic range size (in millions of km^2^) from CIESIN
• population density (millions of people per km^2^) from CIESIN
• a quadratic term for population density, because we reasoned that at very high and very low population densities (i.e., very urban and very rural areas) photos would be rare, in the first case because of habitat destruction and in the second because of little effort
• global region (North America = USA + Canada; Latin America including Mexico, the Caribbean, Central and South America, Oceania including Australia, Papua New Guinea, New Zealand, and Pacific Islands, Asia including the Middle East, the Caucasus, Turkey, Russia east of the Ural mountains, and West Papua; Africa including Madagascar and the Mascarene Islands; and Europe including Russia west of the Ural mountains)
• medically-important venomous snake (MIVS) (yes or no) from the World Health Organization
• blindsnake (yes or no) from the Reptile Database
• year of original description (1758–2020) from the Reptile Database

so the overall model was:

$$ln\left(number\;of\;photos+1\right)\;\sim \;\text{ln}\left(range\;size\right)+\text{ln}\left(population\;density\right)+\text{ln}\left(population\;density^{2}\right)+continent+mivs+blindsnake+year$$

## 3 Results

The number of photos and species obtained from each data source are found in Table 1. Combined, 3098 of the world's 3879 snake species (79.9%) are represented in these eleven data sources by at least one photo (Table 2). This leaves 781 “most wanted” species with no photos (20.1% of all currently-described species as of the 17 December 2020 release of The Reptile Database; see Table 2 for a summary and Appendix 4 for a list sortable by family, continent, authority, and MIVS status).

**Table 1:** Number of photos, number of species, and number of unique species (only from that data source) from each of the data sources.

source	CalPhotos	Flickr	HerpMapper	iNaturalist	ISI	Literature	Private	TRD	Twitter	VMUS	Wikimedia
photos	5,506	56,945	124,378	414,115	5,593	671	56,268	6,686	5,018	48,387	1,998
species	915	1,560	1,127	2,273	5	324	1,788	1948	323	234	1,031
unique species	11	55	10	199	0	159	34	200	1	12	38

**Table 2:** Percent of photos from each global region that come from each of the data sources. iNaturalist contributed the most photos for every global region except Africa. See text for definitions of global regions.

source	Africa	Asia	Australia	Europe	marine	Canada + USA	Latin America
CalPhotos	0.7	2.2	2.2	1.6	1.5	0.2	1.7
Flickr	5.8	19.3	34.2	13.8	14.5	4.4	9.6
HerpMapper	0.8	6.5	3.3	1	2.4	24.8	11
iNaturalist	19.2	40.5	42.5	60.8	55.8	66.6	55.8
ISI	0	8.7	0	0	0	0	0
Literature	0.2	0.4	0.1	0	0.4	0	0.3
Private	5.7	17.2	14.5	20.4	9.5	3	18.3
TRD	1.2	3.3	1.5	0.7	4.7	0.1	2.7
Twitter	0.5	0.7	0.9	1.4	1.5	0.8	0.2
VMUS	65.6	0.1	0	0	7.2	0	0
Wikimedia	0.4	1.2	0.8	0.3	2.5	0.1	0.4

In total we collected 725,565 photos. The largest data source, iNaturalist, made up 57.4% of all photos, whereas the smallest, primary literature, made up just 0.09%, but contributed photos of 163 species found in no other data source (Table 2). More than half of all species (56.7%) were represented by 10 or fewer photos, whereas a small number (12; 0.3%) were represented by > 10,000 photos each (Table 3).

**Table 3:** Number species in each photo bracket. MIVS = medically-important venomous snakes.

Photo bracket	Number of species (MIVS)	Percentage of species	Cumulative percentage
no photos	781 (29)	20.1	20.1
1 photo	379 (43)	9.8	29.9
2-10 photos	1,027 (113)	26.5	56.4
11-100 photos	1,089 (228)	28.1	84.5
101-1000 photos	479 (119)	12.3	96.8
1001–10,000 photos	112 (30)	2.9	99.7
more than 10,000 photos	12 (4)	0.3	100

### 3.1 Geographic distribution of the dataset

**Fig. 3:**
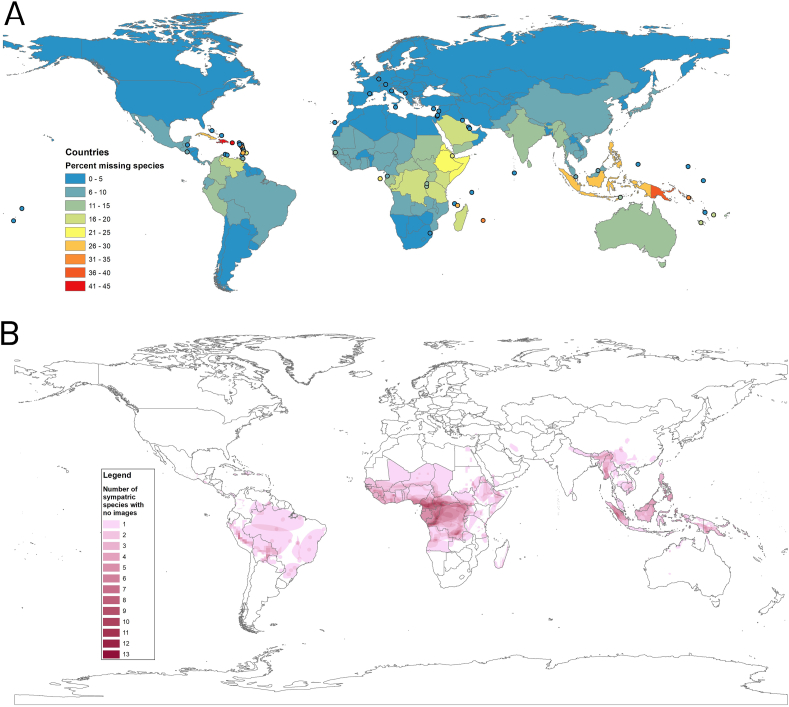
A) Percent of snake species in each country with at least one photo. Ninety-four countries have photos of every species in the dataset. Indonesia, with a high percentage of island endemic species, has 102 snake species (29%) without any photos in the dataset. Globally, the Caribbean, the Horn of Africa, and the islands of southeast Asia are the regions with the greatest need of additional data collection. B) Heatmap of ranges of species with no images, data from GARD (Roll et al., 2017). Photographers with photos of missing species (see Appendix 4) are encouraged to submit them to The Reptile Database.

A total of 107 countries (not including countries that lack snakes entirely, such as Ireland, Iceland, New Zealand, and Cape Verde) have all species represented, mostly in Europe, central Asia, North and Central America, and the Lesser Antilles. Many other countries with complete coverage are relatively small (e.g., Belize, Lebanon, Qatar), insular (e.g., Bahrain, Comoros, Maldives, Nauru, Palau, Seychelles, Tonga, Vanuatu), or occur in more temperate regions with somewhat lower biodiversity (e.g., Chile, North Korea, South Korea, Uruguay). Only a handful of countries in Africa, all from the far north (Morocco, Algeria, Tunisia) and far south (Botswana, South Africa, Lesotho, Eswatini) have 100% of their snake species represented by at least one photo in our dataset (Fig. 3).

**Fig. 4:**
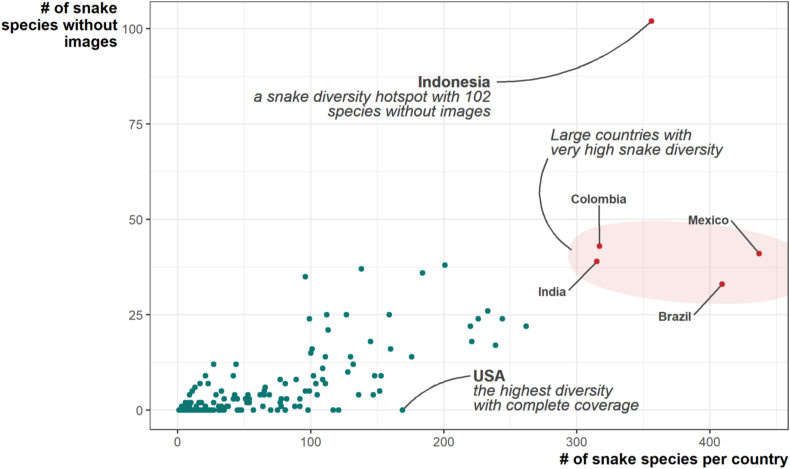
Number of species per country plotted against the number of species with no photos found in that country. Country lists are from the Reptile Database (Uetz et al., 2021).

Globally, the Caribbean, the Horn of Africa, and the islands of Southeast Asia are the regions with the greatest need for additional data collection, although large biodiverse countries that encompass a diversity of ecoregions, such as Australia, Brazil, China, Colombia, India, Indonesia, Mexico and Peru, are priorities because of the enormous number of species found there (Fig. 3). Indonesia, Papua New Guinea, the Philippines, Cuba, Haiti, and the Dominican Republic stand out as having more missing species than expected for their diversity; the USA, Brazil, Mexico, Costa Rica, Ecuador, Nicaragua, Suriname, and Belize have fewer missing species than expected for their diversity (Fig. 4).

Canada and the USA have between 4 and 22 times as many photos as the other global regions, a phenomenon present in citizen science data as a whole (La Sorte and Somveille, 2020). There is a positive non-linear relationship between range size and number of photos in all global regions, especially in North America and Europe. The percentage of photos from each global region varies by data source—iNaturalist, HerpMapper, and Twitter photos come mostly from North America, but many Flickr photos come from Asia, many CalPhotos and private photos come from Latin America, TRD and literature photos are concentrated in Asia, Africa, and Latin America, all ISI photos come from Asia, and all VMUS photos come from Africa (Table 4).

**Table 4:** Percent of photos from each data source that come from each of the global regions. See text for definitions of global regions.

global region	CalPhotos	Flickr	HerpMapper	iNaturalist	ISI	Literature	Private	TRD	Twitter	VMUS
Africa	9.1	7.4	0.5	3.4	0	18.3	7.4	12.9	6.9	99.6
Asia	25.5	21.8	3.3	6.3	100	35	19.6	31.6	9.4	0.2
Australia	8	11.9	0.5	2	0	1.9	5.1	4.6	3.5	0
Europe	10	8.6	0.3	5.2	0	1.5	12.8	3.7	10.1	0
marine	0.4	0.4	0	0.2	0	1	0.3	1.1	0.5	0.2
Canada + USA	17.9	33.5	86.7	69.8	0	1	23.2	6.9	66	0
Latin America	29.1	16.4	8.6	13.1	0	41.1	31.6	39.2	3.6	0

### 3.2 Model

Of 128 explanatory variable combinations tested using the dredge function in MuMIn, the global model had the lowest AIC and 99.99% of the weight, with all terms significant. The second best model had a ΔAIC_c_ of 31 and was missing only the quadratic term for human population density. Thus, we describe the impact of all main effects on the response variable, number of photos per species. For the full model table and detailed parameter estimates from the top model, see Appendices 5 and 6.

#### 3.2.1 Variation among global regions and between blindsnakes and alethinophidians

In descending order, the global regions with the most photos were North America (mean ± S.D. number of photos per species = 2619 ± 5675), Europe (928 ± 2120), Africa (115 ± 387), Australia (88 ± 229), Latin America (77 ± 292), and Asia (62 ± 219). Variation in the maximum number of photos per species was comparable (Table 5). All European snake species and all North American and Australian MIVS were represented by at least 1 photo (Table 5).

**Table 5:** Average, standard deviation, and maximum number of photos per species by global region, and number of species with 0 photos from each global region. See text for definitions of global regions. MIVS = medically-important venomous snakes *most marine snakes are potentially dangerous but rarely bite, whereas 4 species have atrophied fangs and venom glands (Li et al., 2005; Shine et al., 2004).

Global region	Snake group	Number of species	Number of species with no photos	Average number of photos per species	Standard deviation of number of photos per species	Maximum number of photos per species	Total number of images
Africa	blindsnakes	125	72	34	141	1048	4228
	non-MIVS non-blindsnakes	418	67	117	372	3159	48708
	MIVS	92	11	221	605	4491	20325
Asia	blindsnakes	77	47	28	205	1797	2154
	non-MIVS non-blindsnakes	792	181	46	127	1352	36591
	MIVS	148	2	167	458	4850	24745
Australia	blindsnakes	61	23	5	10	54	335
	non-MIVS non-blindsnakes	129	18	92	235	2174	11927
	MIVS	34	0	219	332	1366	7458
Europe	blindsnakes	1	0	14	NA	14	14
	non-MIVS non-blindsnakes	20	0	1309	2619	11840	26186
	MIVS	17	0	534	1358	5415	9082
Canada + USA	blindsnakes	7	1	169	390	1050	1184
	non-MIVS non-blindsnakes	136	7	2550	5883	48696	346760
	MIVS	22	0	3831	5042	14887	84286
Latin America	blindsnakes	126	46	10	49	532	1270
	non-MIVS non-blindsnakes	967	182	72	260	4176	69299
	MIVS	186	9	150	477	3612	27920
marine	non-MIVS*	4	0	21	14	33	83
	MIVS	67	18	22	73	480	1493

Blindsnake species had on average 10 times fewer photos (mean ± S.D. = 23 ± 134 per species) than alethinophidian snake species (mean ± S.D. = 241 ± 1507 per species). Nearly half (189 of 397; 47.6%) of blindsnake species in GARD are represented by 0 photos, in contrast to less than a sixth (477 of 2961; 16.1%) of alethinophidians in GARD. The blindsnake species with the most photos (1,797), *Indotyphlops braminus*, is a globally-distributed parthenogenetic all-female triploid human commensal species (Wallach, 2020) with a range size of 3.3 million km^2^, nearly five times more widely distributed than the blindsnake with the next highest number of photos (*Rena dulcis* with 1050) and the only blindsnake with a range size larger than the average for all snakes (915,000 km^2^).

#### 3.2.2 Effect of range size and human population density on number of photos

**Fig. 5:**
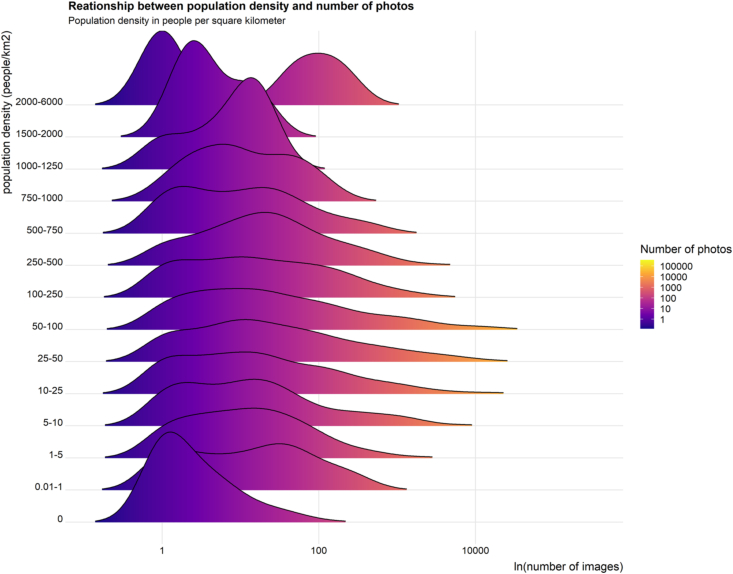
Non-linear relationship between population density (people/km^2^) and number of photos (log). Y-axis breaks are of unequal size to better show the relationship.

There was a positive effect of range size on number of photos (Fig. 2). For every additional 100,000 km^2^ of range area, there were 327 ± 15 more photos. For every additional thousand people per km^2^ within the range, there were 1644 ± 196 more photos (Fig. 2). The sign of the significant quadratic term is negative, supporting our hypothesis that the shape of the relationship is concave down rather than concave up—that is, snakes found in areas with very high and very low population density tend to have fewer photos than those found in areas of intermediate population density (Fig. 5).

#### 3.2.3 Effect of date of description on number of photos

**Fig. 6:**
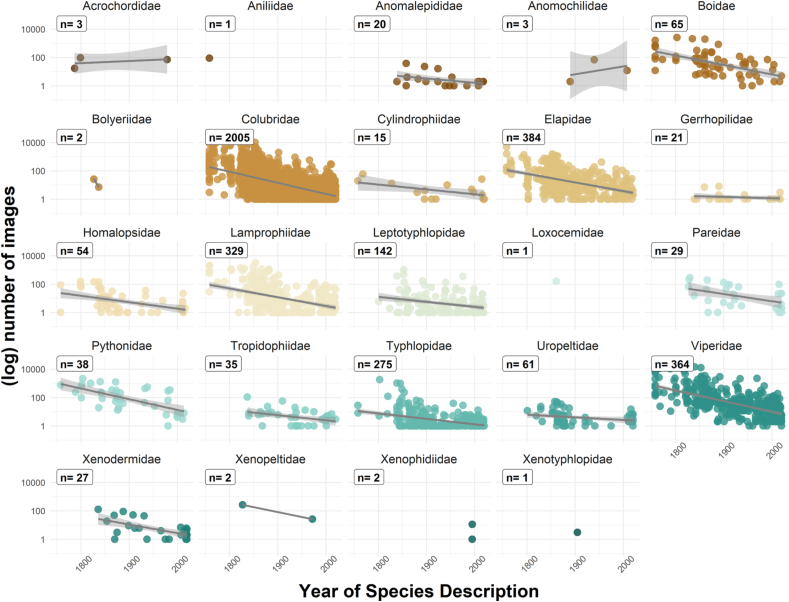
Year of original species description (Uetz, 2010) plotted against the number of photos (log) in our dataset, by family. Each point is one species. Species that were described before 1900 are more likely to have >1000 photos (Spearman's rho = −0.54; see Appendix 5 for parameter estimate). Notably, we could not locate even one photograph in life of one species of snake, *Brachyorrhos albus*, described by Linnaeus in 1758 from Seram, Ambon, and nearby islands in eastern Indonesia (Murphy et al., 2012).

Species described longer ago are represented by more photos (Fig. 6). On average, every year since 1758 corresponds to 1.8 fewer photos. Only three species that were described after 1900 have >1000 photos. No species described since the year 2000 has more than 302 photos (*Contia longicaudae* from the northwestern part of North America). The species with the most photos, *Thamnophis sirtalis*, was described by Linnaeus in 1758. There is a bi-modal distribution in the number of photos, with a peak at 1 and a second peak near 10.

#### 3.2.4 Relationship between number of photos and age of scientific name for recently revalidated species

We used a linear model with the form:

log(numberofimages+1)~log(yearsafter1758+1)+presenceofnameinGARD(y/n)

to ask whether the more widespread *sensu lato* species names or the more restricted *sensu stricto* species name(s) that have been revalidated since the GARD mapping project had more photos. The model had an adjusted R^2^ of 0.33.

An increase in the number of years since 1758 led to a decrease in the number of photos, but we defer to the more complex model for effect sizes. The more widespread *sensu lato* species mapped in GARD had 2.2 ± 0.3% more photos (379 ± 52 more photos) compared to the more restricted *sensu stricto* species that have been revalidated since the GARD mapping project was published in 2017. This effect is significant at p < 0.001. A test removing the most extreme outlier (*Boa orophias*) did not alter the results of the model.

#### 3.2.5 Effect of medical importance on number of photos

Medically-important venomous snakes (MIVS) are represented on average (±S.D.) by 348 ± 1385 photos, whereas non-medically-important species are represented on average (±S.D.) by 192 ± 1422 photos. Only 22 of the 499 MIVS species in GARD (4.5%) are represented by 0 photos, whereas 644 of the 2859 non-MIVS species in GARD (22.5%) are represented by 0 photos (decreasing to 455 [18.5%] when only the 2462 non-blindsnakes are considered). This is despite the fact that six non-MIVS species are represented by more photos than the MIVS species with the highest number of photos (*Crotalus oreganus* with 14,831).

### 3.3 Growth rates of selected data sources

**Fig. 7:**
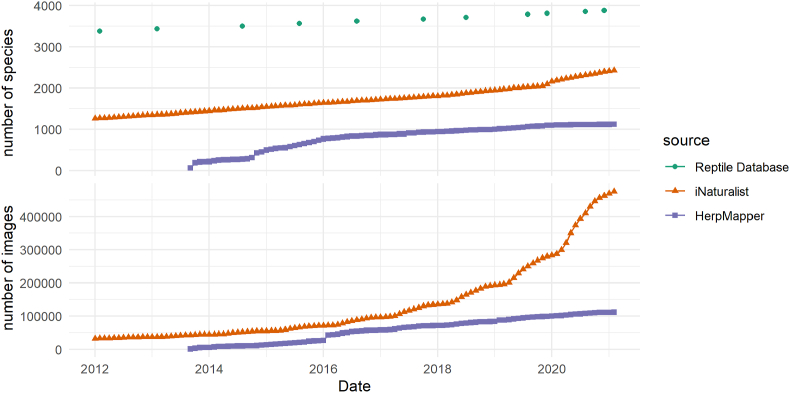
Growth of iNaturalist & HerpMapper datasets over time, with corresponding increase in number of described snake species (each point is one Reptile Database release; Uetz et al., 2021).

As of 30 March 2021, the number of observations of snakes in iNaturalist is 487,948 (many with >1 photo, see above) and the number in HerpMapper is 124,378, with average annual growth from 2018 to 2020 of 49% and 18%, respectively. The rate of growth of iNaturalist is accelerating whereas that of HerpMapper is steady (Fig. 7).

## 4 Discussion

### 4.1 Overview

We present a summary of >700,000 photos of nearly 80% of snake species worldwide, adding new datasets, species, and photos to the recent compilation for all reptiles (Marshall et al., 2020a). Overall, our spatial and taxonomic coverage were comparable, and we expect that further effort would result in greater species coverage, but with ever-diminishing returns.

North America is over-represented in nearly all citizen science projects, because it is where many of those projects began and are based, and many North Americans have substantial recreational and leisure time to contribute observations compared to many people living in developing countries (Millar et al., 2019), who likely have different priorities, different interests, different beliefs about touching animals and taking photos (Hauenstein, 1978), and poorer access to cameras and broadband (although this is improving rapidly even in remote areas; Broadband Commission, 2019; James, 2020). Spatial bias is important to account for when using crowd-sourced data to map species distributions (Johnston et al., 2020). High-income countries supply most snake observations in citizen science platforms, whereas countries with the highest snakebite burden have much more limited participation (Geneviève et al., 2018).

Blindsnake species are inherently difficult to detect and identify, even more so than snakes in general. Most blindsnakes are tiny (<30 cm), spend most of their time underground, and thus are easily overlooked. Furthermore, even experts have difficulty identifying blindsnakes to species without counting their scales. Thus, it is not surprising that they are less likely to be represented by photos than other snakes.

### 4.2 Model

#### 4.2.1 Effect of range size and human population density on number of photos

There is a significant effect of range size. Range size is dependent on a number of factors, including biogeographic history, habitat suitability, dispersal capability, taxon age, and species delimitation, and there is concern that common techniques in molecular phylogenetics might over-split species (Chambers and Hillis, 2020; Freitas et al., 2020; Hillis, 2019), which can lead to inconsistent application of species definitions (and thus range size) throughout the world (see next section). It is unsurprising that snake species endemic to a small area are less likely to be represented by photos in our dataset, but we emphasize that many snake species once thought to be known only from the type locality have since been proven to be much more widespread (e.g., Mebert et al., 2020).

We found support for a non-linear influence of human population density on photo number. The 26 snake species with >5000 photos in the dataset were all found in areas between 6 and 86 people/km^2^. Of snake species that occur in areas with <1 person/km^2^, 43% had 0 photos, and 72% had <5 photos. However, some impressive outliers also existed. Five snake species remain sufficiently abundant in areas of relatively high human impact (population density >750 people/km^2^) to be represented by > 50 photos (*Chilabothrus inornatus* from Puerto Rico, *Naja sputatrix* from Java, Bali, and adjacent islands, *Opisthotropis andersonii* from Hong Kong and Vietnam, *Sinomicrurus hatori* from northeastern Taiwan, and *Thamnophis scaliger* from the southern part of the Mexican plateau in the vicinity of Mexico City). On the other end of the spectrum, six snake species are sufficiently well-known to be represented by > 50 photos in the dataset despite occurring in areas where the human population density is < 0.05 people/km^2^ (*Acanthophis pyrrhus* from central and northwestern Australia, *Dipsas articulata* and *Tantilla hendersoni* from rural parts of Central America, *Crotalus catalinensis* from the Isla Santa Catalina in the Gulf of California, Mexico, and *Macrocalamus chanardi* and *M. gentingensis* from peninsular Malaysia).

#### 4.2.2 Effect of date of description on number of photos

That species described longer ago have more photos is not very surprising, although it emphasizes how much we have left to learn about many snake species, particularly in developing countries. The number of new snake species described in the 21st century so far has averaged 33 per year, higher than at any other time except the 1860s, when colonialism was at its peak. It is now routine for new species descriptions to contain at least one and sometimes several color photographs, but this has only become possible within the past few decades. Notably, we could not locate even one photograph in life of one species of snake, *Brachyorrhos albus*, described by Linnaeus in 1758 from Seram, Ambon, and nearby islands in eastern Indonesia (Murphy et al., 2012). More recently described species can lack photos in life if they are described from museum specimens.

Only a few species that were described after 1900 have >1000 photos, and all of these are North American species that were re-elevated from more widespread species relatively recently (*Agkistrodon conanti*, *A. laticinctus*, *Lampropeltis holbrooki*). We attempted to test whether the photo dataset is biased towards older names when species have been recently split, as described by Marshall et al. (2020), because we had the impression that when widespread species are split or elevated from subspecies to species, creating new binomials, originally correct identifications can become incorrect. We found evidence that this is the case—the more widespread *sensu lato* species mapped in GARD had almost 400 more photos compared to the more restricted *sensu stricto* species name that has been revalidated since the GARD mapping project was published in 2017. However, the tangled taxonomic history of particular names can obscure this problem—for instance, a major outlier is *Boa orophias*, the St. Lucia boa, originally described as a full species by Linnaeus in 1758 but repeatedly upgraded and downgraded to a subspecies of *Boa constrictor* (Reynolds and Henderson, 2018) until the most recent elevation (Bezerra de Lima, 2016). We found only 11 photos labaled as *Boa orophias*, partly attributable to its small and insular range, but there are undoubtedly others in the dataset that are labeled as *Boa constrictor*. Even widespread recently-revalidated species that were initially described many years ago, such as *Boa imperator* (also from *Boa constrictor*), *Agkistrodon conanti* (from *A. piscivorus*), *A. laticinctus* (from *A. contortrix*), *Natrix helvetica* (from *N. natrix*), *Naja subfulva* (from *N. melanoleuca*) and *Crotalus pyrrhus* (from *C. mitchellii*) are susceptible to this problem, and names that are very recent are rarely used even when they have been applied to large areas (e.g., *Naja savannula* in west Africa; Wüster et al., 2018).

Taxonomic uncertainty in some of the major groups of phenotypically-similar medically-important venomous snakes (e.g., *Bungarus*, Sunagar et al., 2021; *Echis*, Trape, 2018) makes this an especially relevant problem for public health (Carrasco et al., 2016; Williams et al., 2006), because antivenoms must be manufactured against specific lineages that vary in their venom composition. Rigorous tracking of locations and photographs that allow reassessment of key characters can help mitigate the identification issues caused by species splits. Because iNaturalist makes regular updates to geo-tagged observations in case of taxonomic changes (https://www.inaturalist.org/taxon_changes) and makes up the majority of our dataset, this problem is not as bad as it might be, but non-geo-tagged data sources (e.g., Flickr, CalPhotos, photos in private collections) are probably very susceptible.

#### 4.2.3 Effect of medical importance on number of photos

There is a significant effect of MIVS status. This could reflect a bias towards reporting medically-important species, that these species are more abundant, more detectable, and/or that their ranges include higher levels of participation in citizen science. Many people are fascinated by venomous snakes (Roll et al., 2016), but in most areas MIVS are not more abundant or diverse than non-MIVS (Luiselli et al., 2020). Citizen scientists in the Carolinas were more likely to submit photos of snakes than of other reptiles and amphibians (Price and Dorcas, 2011), suggesting that fascination with or fear of snakes might motivate people to document them at higher rates than other taxa, but whether this is particularly true for venomous snakes remains to be rigorously tested (and in many cases the citizen scientist submitting the photo might not know whether the snake is venomous or not).

### 4.3 Growth rates of selected data sources

Linear extrapolation of slopes of species accumulation curves suggests that observations of previously-unreported species are added to online biodiversity platforms more quickly (iNaturalist: 9.5 new species per month; HerpMapper: 12.3 new species per month) than the current rate of new species description (4.9 new species per month) and that online biodiversity platforms might contain at least one photo of every species as early as 2050, although it is more likely that all three of these curves will begin to asymptote during the 21st century, causing a persistent deficit of rare species. Obviously such simplistic extrapolations of future trends in species description and citizen science participation are highly uncertain and likely to be influenced by a variety of unforeseeable forces. For example, Hochachka et al. (2021) documented regional variation in the impacts of the COVID-19 pandemic on the quantity and quality of data collected by the citizen science project eBird, and we expect that social, economic, and political factors play a large role in which species are most well-documented in online biodiversity databases.

### 4.4 Limitations of the different data sources

We highlight six limitations of the dataset:

1. All data sources contain some **misidentified photos.** A crowd-sourced review in which an average of 110 participants identified each photo (min 68, max 156) found that five (1.5%) of 336 Research Grade photos of snakes from iNaturalist were incorrectly identified (Durso et al., 2021a); likewise, 16 of 703 photos (2.3%) reviewed from 20 Snake ID Facebook groups were incorrectly identified. Most of these were due to recent taxonomic changes or confusion with similar species. If we assume similar error rates for our other data sources, there could be as many as 13,700 incorrectly identified photos in our dataset, although platforms without formal species identification curations (e.g., Flickr) likely have higher misidentification rates. Finding and correcting these is a priority, as is keeping a record of the misidentification and its repair. iNaturalist in particular does an admirable job of showing the annotated identification history in a transparent way.
2. Using a **consistent taxonomy** across data sources would make de-siloing and connecting photos of the same species that are tagged using different names much easier. No single part of this project was more time-consuming and frustrating than trying to accomplish this in a repeatable, documented, and accurate way. This problem is not unique to photo data, and scientists as diverse as geneticists and plant ecologists (Boyle et al., 2013; Garg et al., 2019; Patterson et al., 2016; Tedersoo et al., 2015) have highlighted this critical challenge. Weiser et al. (2007) compiled >300,000 plant observations from 51 sources, resulting in >22,000 unique names, which fell to 12,980 after correcting misspellings and updating synonymous names—that is, >42% of the names in the original data were erroneous, obsolete, or otherwise inconsistent with the then-currently accepted names (and even a one-time effort to correct such names will drift out-of-date as new taxonomic changes are made)! Our experience collecting this dataset suggests that the problem for snakes is of a similar magnitude and, left unaddressed, represents an insidious source of error. Adherence to DarwinCore standards (Wieczorek et al., 2012) is a good start, as is the R package taxize (Chamberlain et al., 2019), although we found that even using these tools resulted in a substantial number of names that remained unmatched and had to be connected using custom dictionaries created manually.
3. **Over- and under-representation.** Some data sources, particularly Flickr, contain many photos that show only the habitat, without an actual snake, despite being tagged with a snake name. A similar attempt to scrape plant biodiversity data from Flickr (August et al., 2020) found that photos were **spatially aggregated** around tourist sites and under-represented native species, and that photos focused on a single individual were most reliably identified by computer vision approaches. They also recommend attempting to quantify the degree to which **charismatic species** are over-represented or **nocturnal species** under-represented, and that adequately documenting procedures for data collection and filtering will be essential for meeting data standards for biodiversity photo datasets which have not yet been formalized.
4. Our experience collecting photos from **Twitter** was less fruitful than hoped. This data source contained many duplicates, even after targeted attempts to remove them. A close look at the photos for a species (*Atheris hispida*) where Twitter photos made up a high proportion (35%) of the total photos in the dataset revealed that numerous duplicate photos remained, despite our efforts to filter them out in advance. Differences in photo size, format (.png vs.jpg), cropping, saturation, and other attributes that cause identical or near-identical photos to appear different to a computer, combined with the pulsed, real-time nature of Twitter content, probably account for most of these duplicates. Another limitation is that only a small number of the photos (289; 0.1%) had geographically-tagged data, without which it is often difficult to confirm the identity. In addition, examining the three species (one photo each) that were added to the dataset exclusively through Twitter revealed that two of these were photos that came from recently-published and publicized original descriptions (Jins et al., 2018; Rödel et al., 2019), where they would have been easy to find had we looked there first. The third was a photo of a nearly extinct species, *Erythrolamprus ornatus*, taken by a Fauna & Flora International (FFI) photographer on Maria Major Island off the coast of St. Lucia in the West Indies, which may be represented by fewer than 20 wild individuals (Williams et al., 2016). This last species is the only one that would not have been included through our other sources, and led to additional photos of this and other rare species when we contacted FFI. Although these gains are undoubtedly important, we suggest that the cost:benefit of Twitter photos is among the lowest of the data sources we attempted. We anticipate that other popular online photo-sharing platforms, such as Instagram or Pinterest, would suffer from the same problem, and are even more likely to have had the color or other attributes altered or to show snakes in captivity. The only exceptions are the highly-curated Facebook snake ID groups (see appendix of Durso et al., 2021a for a list), which are essentially impossible to access in a scalable, reproducible way due to Facebook's policies (i.e., required use of personal access points, and removal of Exchangeable Image File [exif] data from uploaded images).
5. All of our data sources are **geographically biased** towards places where people with access to technology (e.g., GPS enabled devices, and citizen science platform language localization) are likely to go. When Marshall and Strine (2019) incorporated incidental occurrence records extracted from geo-tagged photos from Flickr into distribution models, they achieved only negligible differences in species distribution model performance, due in part to the disproportionate origin of these records being from parts of the world that are already well-sampled. Furthermore, globally >750 million people (11% of those living in areas with medically-important venomous snake) live >1 h from population centers, mostly in sub-Saharan Africa, Indonesia, and other parts of southeast Asia where our data are also the most incomplete (Longbottom et al., 2018). Finally, biodiversity of snakes is higher in the tropics (Roll et al., 2017), but species evenness is also higher (Luiselli et al., 2020), such that more rare species exist in the tropics, which (in addition to differences in human development and technological access between temperate and tropical zones) probably partially explains the higher number of missing species in tropical countries (Fig. 3).
6. We did not make an effort to collect images that could only be identified to a **higher taxonomic category** (e.g., genus or family), which can happen due to the inability to see important characteristics in the image, but these represent a way to represent uncertainty in species-level identification. Many photos can only be reliably identified to the genus or family level, yet this still represents information about the category to which the animal in the photo belongs even if a precise species-level identification cannot be made. Incorporating such nested hierarchical uncertainty in identification is challenging but possible (Durso et al., 2021a), similar to genetic techniques for narrowing down the identity of a DNA sequence (e.g., Singh et al., 1999).

### 4.5 How to improve in the future

We outline four primary directions of improvement:

1. **Continue growth of existing datasets**. The rate of accumulation of new species and photos in iNaturalist and HerpMapper is promising. Promisingly, citizen scientists in the Carolinas were more likely to submit photos of snakes than of other reptiles and amphibians (Price and Dorcas, 2011), suggesting that fascination with or fear of snakes might motivate people to document them at higher rates than other taxa, even though they are probably encountered at lower rates. Given that most people rarely encounter snakes due to their cryptic habits (Dorcas and Willson, 2009), a snake encounter might also seem more worthy of documentation. Outreach campaign at universities and institutions, particularly in developing countries, encouraging the use of citizen science platforms for documenting snakes would be very valuable. By comparison, citizen science databases on birds dwarf those on reptiles. Citizen data targeting birds demonstrate what is possible regarding geographic and species coverage, while simultaneously collecting important survey metadata (metrics to quantify effort), and stimulating prolonged public engagement (Johnston et al., 2019; La Sorte and Somveille, 2020). For example, the Cornell Lab of Ornithology Macaulay Library contains over 24 million photos covering 10,046 of 10,721 (93.7%) of the world's species of birds, and the total number of eBird observations (including those without photos) is over 950 million. On the other hand, fundamental differences between the behavior and ecology of birds and reptiles, as well as the susceptibility of some reptile populations to exploitation and the necessity of protecting sensitive locality information, may limit what is possible when citizen scientists gather and report data on reptiles. Illegal trade in snakes and snake parts is thriving in some regions (Hierink et al., 2020; Marshall et al., 2020b) and many herpetologists are understandably reluctant to divulge geographic location information that might compromise wild populations. On the other hand, the frequency and impact of the illegal trade can be overblown, which can hinder attempts to obtain core data on images and permits for biological field work, and divert attention from other, more destructive threats (e.g., Mebert et al., 2020).
2. **Untapped non-peer-reviewed online datasets**. Other than simply waiting for iNaturalist and HerpMapper to continue to grow, the next best way to enlarge this dataset would be to query difficult to access sub-communities such as Facebook snake identification groups. In contrast to Twitter, most of the non-snake, viral, duplicate, or otherwise irrelevant photos have already been filtered out of these collections by diligent moderators and administrators, and every photo contains a species common and/or scientific name in the caption or comment thread. Other social media platforms, such as Baidu Tieba (https://tieba.baidu.com/f?kw=%C9%DF&fr=ala0&tpl=5), also host snake-specific sub-forums that likely contain numerous photos that are paired with accurate identifications. The Field Herp Forum (http://www.fieldherpforum.com/forum/index.php) is a reptile and amphibian specific forum where users post photos of reptiles together with descriptions of their field experiences searching for and photographing them. This community places a high value on protecting locations that represent excellent snake habitat from becoming public knowledge, because of fears that well-meaning but inexperienced enthusiasts may degrade those habitats in their search for wild reptiles, or that reptiles may be collected from the wild for sale into the pet trade. Consequently, Field Herp Forum is unlikely to be a source of geo-tagged photos. Finally, there are numerous private WhatsApp groups that are used for snake identification. One such group to which the lead author belongs is focused on southern Africa and routinely identifies 5–10 snake photos per week during the active season. Seasonal variation in the number of new ID requests (and thus photos) is also apparent in Facebook snake ID groups (e.g., 10 per day in Northern Hemisphere winter vs. >300 per day in Northern Hemisphere spring; Smith et al., 2019) as well as in iNaturalist and HerpMapper data (Fig. 7). In general, citizen scientists equipped with the infrastructure to follow good practices are likely to continue to lead to new discoveries in biodiversity and help flesh out our understanding of the distribution of snakes and other animals (Liberatore et al., 2018).
3. **Untapped offline datasets**. Many valuable photos are published in books, which are subject to copyright. A painstaking search through the primary literature would likely reveal additional such photos, but would necessarily have to proceed arduously and by hand. Additionally, many such photos may be of poor quality, and may bear out-of-date names that will be difficult to connect with modern taxonomy unless the geographic provenance of the individual in the photo is known (Simkins et al., 2020). Additionally, many natural history museums have slide collections that contain hundreds to tens of thousands of photos of snakes, many of which are linked to specimens, but resources to digitize these collections are largely lacking. We expected to find more of these in GBIF. We did not focus on collecting photos of preserved specimens, but some species are undoubtedly represented only by such photos at present. The utility of such photos is limited with respect to natural coloration and often geographic location, but they allow experts to focus on critical (diagnostic) characters and gathering these, especially of holotypes, would certainly have value. We also did not focus on gathering photos that could be identified only to the genus or higher taxonomic level, photos of snakes in captivity (including captive-bred color morphs), or photos of captive-bred or naturally-occurring hybrids (LeClere et al., 2012; Mebert, 2008). Aberrant coloration and patterning occurs at low frequencies in wild snakes (Borteiro et al., 2021), and individual, regional, ontogenetic, and coloration and patterning are common (Bechtel, 1978; Farooq and Uetz, 2020), so continuing to collect photos even of common species has value.
4. **Peer-reviewed literature**. After combining data from our first 12 sources, we conducted targeted searches of the peer-reviewed literature (mostly original descriptions) for species with 0 photos. This was very effective (5.3% of species added this way) but returns diminished over time. Furthermore, 12.3% of species are represented in the dataset by only a single photo. Bringing these species above even a modest threshold (e.g., 10 photos) would undoubtedly require more of the same targeted literature searching, plus field expeditions to relocate, photograph, and clarify the status of species not seen in some cases for many decades (e.g., Andreone and Raxworthy, 1998; Gower et al., 2004; Lanza, 1990; Mulcahy et al., 2014; Murphy et al., 2008; Passos et al., 2009; Ramírez-Bautista et al., 2013; Rasmussen et al., 2012).

### 4.6 Very rare, obscure, or probably extinct species

Many species of snakes are so rarely seen that any new specimen is worthy of publication (e.g., Bauer et al., 2001; Fukuyama et al., 2020; Murthy, 1985; Ota and Mori, 1985; Smaga et al., 2019; Stuebing and Goh, 1993; Yaakob, 2003). At least 52 snake species (1.3%) are known from just a single specimen (Wallach et al., 2014). Some of these species have extremely unusual, mysterious, or tragic origin stories. For example, both *Geophis dunni* and *Cenapsis aenigma* were discovered within the digestive tracts of *Micrurus* coralsnakes, and have never been seen in other contexts. *Argyrogena vittacaudata* and *Epictia undecimstriata* were each described from a single specimen, both of which have been lost. The type specimens of about 320 snake species are either lost or their whereabouts are unknown (Uetz et al., 2019), significantly more than among lizards, and this reflects their relative rarity. The holotype and only known specimen of *Anoplohydrus aemulans*, a monotypic genus, was destroyed in July 1943 during the bombing of Hamburg in WWII, along with both known specimens of *Typhlops hypsobothrius*. The tragic fires at the Museu Nacional de História Natural e da Ciência in Lisbon in 1978 and at the Instituto Butantan in São Paulo in 2010 (De Lima, 2010; Franco, 2012; Warrell et al., 2010) destroyed the holotypes and only known specimens of *Tricheilostoma dissimilis* and *Phalotris concolor*, respectively.

The geographic origin of the single specimen of *Cathetorhinus melanocephalus* is unknown (Cheke, 2010; Wallach and Pauwels, 2008). An unfortunate number of species, including *Borikenophis sanctaecrucis* (from St. Croix, U.S. Virgin Islands), *Clelia errabunda* (from St. Lucia), *Erythrolamprus perfuscus* (from Barbados), *Hypsirhynchus melanichnus* (from Hispaniola), and *Bolyeria multocarinata* and *Madatyphlops cariei* (from Mauritius) are probably extinct, the last known only from subfossil remains (Arnold, 1980; Cheke, 1987; Henderson, 1992; Korsós and Trócsányi, 2006). Photographs of these species in life are not likely to be forthcoming in the near future, and even some of their genus allocations are speculative. Of course, digitization of data on the world's natural history museum collections is an ongoing process and we anticipate that some of these species may be “rediscovered” (and that undescribed species still lurk in undigitized, unexamined museum jars; e.g., Kaiser et al., 2020; Kieckbusch et al., 2016; O'Shea et al., 2020).

### 4.7 Potential applications of snake photos

We collected these photos because we foresee a variety of potential applications:

1. As **validation of occurrence** records, which rapidly increase our knowledge of the distribution of species, particularly species of conservation concern, and to track climate-induced shifts in geographic distribution (Yañez-Arenas et al., 2016; Zacarias and Loyola, 2019) and the spread of non-native species (Gray, 2020; Mo, 2019; Montes et al., 2021). However, photos can only serve as documentation of the presence of a species, whereas absence/non-detection is also important to document, especially for cryptic species (O'Donnell and Durso, 2014). Furthermore, online photo data evaluation can **impact systematics and taxonomy** by enabling more precise location of contact zones between species and initiating investigations of gene flow between taxa (Hillis, 2020; Hofmann et al., 2018; Mebert et al., 2020). However, we caution that uncritical acceptance of geographic locality information, or failure to deposit photos in permanent repositories (such as GBIF or iNaturalist, not Facebook), can result in erroneous records that migrate uncritically into databases and are then almost impossible to correct (e.g., Wangyal et al., 2020 reports a 2500 km range extension for *Oligodon chinensis* but the published photo is actually *O. juglandifer*, and a 2100 km range extension reported for *O. venustus* is not supported with a photo).
2. As data sources in **ecological analyses** (e.g., Todd et al., 2016). Notably, occurrence records that are not associated with a photograph are likely to contain misidentifications and should be used for macro-ecological studies only with caution. Recently social media images/observations have provided insight into the diets of snakes (Maritz and Maritz, 2020), and for other groups (damselflies), community images revealed previously unquantified patterns in phenotypic variation (Drury et al., 2019). Wide-ranging species with hundreds or thousands of images often show geographic variation that may reflect evolutionary gradients and adaptations to changing environments. While herpetologists in the past had only museum collections to work with, such studies may be more powerful if they also use citizen science data with verified digitized location data.
3. As reference material for **new species descriptions**, and for use in **illustrating field guides and identification** keys (Kirchoff et al., 2011; Leggett and Kirchoff, 2011), including digital, interactive, multiple-entry-point keys that should be easier to use with minimal training (Farnsworth et al., 2013; Hsu et al., 2017). Although the gold standard will always be keys that rely on conserved morphological characteristics such as scale counts (e.g., Hsu et al., 2017; Meirte, 1992), simpler keys that take inputs that most users can accurately assess without the benefit of specialized training have been developed and used successfully for other taxa (Boho et al., 2020; Van Horn et al., 2015), using combination of crowd-sourcing and computer vision to guide users to the most likely identifications (Barry, 2016). Farooq and Uetz (2020) found that for most parts of Australia, color, pattern, size and location are sufficient to narrow down the number of possible snake species to fewer than 21.
4. As training material for humans to improve their **snake identification skills** (Kirchoff et al., 2014). There is an urgent need for training more biologists who have expertise in species identification, because such skills have become rare, partly due to a lack of funding for natural history research (Greene, 2005; Kim and Byrne, 2006). Photo collections can be useful supplements to field experience and specimen-based identification courses or labs (Meagher et al., 2018).
5. As training and testing material for **computer vision algorithms** that can assist humans in species identification, including providing an offline benchmark for algorithm performance (Bloch et al., 2020; Durso et al., 2021b; Moorthy, 2020; Picek et al., 2020) as well as supplementing the suggested identification so that the human can compare their unknown snake with the computer's suggestions. This approach has shown great promise for rapidly and accurately identifying other groups of organisms (Seeland et al., 2019; Sulc et al., 2020; Wäldchen and Mäder, 2018; Weinstein, 2018), particularly when combined with high-quality metadata (Leipzig et al., 2021; Valan et al., 2021) and following taxon-specific best practices for acquiring images (Rzanny et al., 2017, 2019).
6. As reference material to use in identifying snakes in **snakebite cases**. The biting snake is never identified in almost half of snakebite cases worldwide (Bolon et al., 2020), including in most of the >6000 snakebites/year at Doctors Without Borders (Médecins Sans Frontières) field hospitals in South Sudan, Ethiopia, the Central African Republic, and Yemen but also in the United States (Ruha et al., 2017), Australia (Johnston et al., 2017), and Europe (Chippaux, 2012). Snakes are seen by snakebite victims in ~70% of cases globally and captured/killed in ~18% of cases (Bolon et al., 2020). Misidentifications can lead to inadequate victim management and can obscure trends in epidemiological data that could otherwise be used to assess how the efficacy of antivenoms and other supportive treatment varies among snake species. For example, two antivenoms are available to treat bites from the 27 species of North American pit vipers, but we know little about whether variation in the choice of snake species venoms used to produce them translates into variation in efficacy in a clinical setting (Cocchio et al., 2020; Dietrich et al., 2019; Gerardo et al., 2017) and we are unable to rigorously test hypotheses because 81% of the time rattlesnake bites are not reported to the species level (Ruha et al., 2017). Mismatches between the species used to produce antivenoms and those responsible for the bites they are used to treat cause wasted antivenom, high costs for patients, morbidity, and mortality worldwide (Rogalski et al., 2017; Senji Laxme et al., 2019; Warrell, 2008). Finally, mapping venomous snake distribution and snakebite risk can aid decisions about the distribution of healthcare resources, snakebite training, and antivenom (Longbottom et al., 2018; Schneider et al., 2021; Yousefi et al., 2020).

## 5 Conclusions

Here we review the status of online snake photos, assemble the largest data set to date covering nearly 80% of all species, and examine some of the biases and pathways to improvement.

In general, scientists should not blindly use data from any source for research purposes, without verifying the accuracy of at least a subset of the identifications and following up on outlier observations (*e.g.,* geographically out of range). All biodiversity databases contain a large number of errors, because natural history collections are underfunded and understaffed (Salvador and Cunha, 2020). We attempted to minimize these, but a large number of incorrectly-identified photos certainly still exist within the data. Large-scale data validation via crowd-sourcing offers one possible solution (Durso et al., 2021a).

Overcoming data deficiency of snake biodiversity is essential for two primary purposes. The first is snake conservation, which is hindered by incomplete knowledge (Bland and Böhm, 2016; Böhm et al., 2013; Reading et al., 2010; Tingley et al., 2016; Tolley et al., 2016). The second has to do with human health, both in terms of risk from venomous snakebite (Geneviève et al., 2018; Longbottom et al., 2018; Williams et al., 2019) as well as novel drug development from venom toxins (Modahl et al., 2019; Modahl and Mackessy, 2019). As we enter the third decade of the 21st century, we call on herpetologists and data scientists to build on the momentum created by the World Health Organization (Williams et al., 2019) to tackle venomous snakebite by engaging with the global health community and continuing to improve the scope, quality, and flexibility of online snake biodiversity resources and imagery.

## Declaration of competing interest

The authors declare that they have no known competing financial interests or personal relationships that could have appeared to influence the work reported in this paper.

## Author credit

**Andrew M. Durso:** Conceptualization, Methodology, Software, Validation, Formal analysis, Investigation, Resources, Data Curation, Writing - Original Draft, Writing - Review & Editing, Visualization, Supervision, Project administration, Funding acquisition. **Rafael Ruiz de Castañeda:** Conceptualization, Methodology, Resources, Writing - Review & Editing, Supervision, Project administration, Funding acquisition. **Camille Montalcini:** Methodology, Software, Validation, Formal analysis, Investigation, Resources, Data Curation, Writing - Review & Editing. **M. Rosa Mondardini:** Conceptualization, Methodology, Resources, Writing - Review & Editing. **Jose L. Fernandez-Marques:** Conceptualization, Methodology, Resources, Writing - Review & Editing. **François Gray:** Conceptualization, Methodology, Resources, Writing - Review & Editing. **Martin M. Müller:** Methodology, Software, Validation, Formal analysis, Investigation, Resources, Data Curation, Writing - Review & Editing. **Peter Uetz:** Conceptualization, Software, Validation, Investigation, Data Curation, Writing - Review & Editing. **Benjamin M. Marshall:** Methodology, Software, Validation, Formal analysis, Investigation, Data Curation, Writing - Review & Editing, Visualization. **Russell J. Gray:** Methodology, Software, Validation, Resources, Data Curation, Writing - Review & Editing, Visualization. **Christopher E. Smith:** Resources, Data Curation, Writing - Review & Editing. **Donald Becker:** Methodology, Software, Validation, Formal analysis, Investigation, Resources, Data Curation, Writing - Review & Editing. **Michael Pingleton:** Resources, Data Curation, Writing - Review & Editing. **Jose Louies:** Resources, Data Curation, Writing - Review & Editing. **Arthur D. Abegg:** Resources, Data Curation, Writing - Review & Editing. **Jeannot Akuboy:** Resources, Data Curation, Writing - Review & Editing. **Gabriel Alcoba:** Conceptualization, Investigation, Resources, Data Curation, Writing - Review & Editing. **Jennifer C. Daltry:** Resources, Data Curation, Writing - Review & Editing. **Omar M. Entiauspe-Neto:** Resources, Data Curation, Writing - Review & Editing. **Paul Freed:** Resources, Data Curation, Writing - Review & Editing. **Marco Antonio de Freitas:** Resources, Data Curation, Writing - Review & Editing. **Xavier Glaudas:** Resources, Data Curation, Writing - Review & Editing. **Song Huang:** Resources, Data Curation, Writing - Review & Editing. **Tianqi Huang:** Resources, Data Curation, Writing - Review & Editing. **Yatin Kalki:** Resources, Data Curation, Writing - Review & Editing. **Yosuke Kojima:** Resources, Data Curation, Writing - Review & Editing. **Anne Laudisoit:** Resources, Data Curation, Writing - Review & Editing. **Kul Prasad Limbu:** Resources, Data Curation, Writing - Review & Editing. **José G. Martínez-Fonseca:** Resources, Data Curation, Writing - Review & Editing. **Konrad Mebert:** Resources, Data Curation, Writing - Review & Editing. **Mark-Oliver Rödel:** Resources, Data Curation, Writing - Review & Editing. **Sara Ruane:** Resources, Data Curation, Writing - Review & Editing. **Manuel Ruedi:** Resources, Data Curation, Writing - Review & Editing. **Andreas Schmitz:** Conceptualization, Resources, Data Curation, Writing - Review & Editing. **Sarah A. Tatum:** Resources, Data Curation, Writing - Review & Editing. **Frank Tillack:** Resources, Data Curation, Writing - Review & Editing. **Avinash Visvanathan:** Resources, Data Curation, Writing - Review & Editing. **Wolfgang Wüster:** Conceptualization, Resources, Data Curation, Writing - Review & Editing. **Isabelle Bolon:** Conceptualization, Methodology, Investigation, Resources, Data Curation, Writing - Review & Editing, Supervision, Project administration, Funding acquisition.
