# Supplementary material for: Citizen science and online data: Opportunities and challenges for snake ecology and action against snakebite
Source: Toxicon X. 2021 Jun 22;9-10:100071. doi: 10.1016/j.toxcx.2021.100071 (PMC8264216; doi:10.1016/j.toxcx.2021.100071)
Supplement: Multimedia component 1 [file mmc1.docx]

Appendix 1: Snake family names and synonyms used to query CalPhotos. Many of these names have also been used at the subfamily level (-inae instead of –idea ending). Some are alternate spellings.

Acrochordidae

Aniliidae

Anomalepididae

Anomochilidae

Aparallactidae

Atractaspididae

Azemiopidae

Boidae

Bolyeriidae

Calabariidae

Calamariidae

Candoiidae

Colubridae

Crotalidae

Cylindrophiidae

Dipsadidae

Elapidae

Erycidae

Gerrhopilidae

Grayiidae

Homalopsidae

Hydrophiidae

Lamprophiidae

Leptotyphlopidae

Loxocemidae

Natricidae

Pareatidae

Pareidae

Prosymnidae

Psammophiidae

Pseudaspididae

Pseudoxenodontidae

Pseudoxyrhophiidae

Pythonidae

Sanziniidae

Sibynophiidae

Tropidophiidae

Typhlopidae

Ungaliophiidae

Uropeltidae

Viperidae

Xenodermatidae

Xenodermidae

Xenopeltidae

Xenophiidae

Xenotyphlopidae

Appendix 2: Search terms used to query Twitter, and search terms considered but not used.

Substrings used (number of snake genera matching the substring, 24 April 2019 Reptile Database release):

ophis (111)

typhlops (18)

elaps (13)

natrix (3)

viper (3)

coluber (2)

echis (6)

elaphe (3)

python (3)

boa (6)

naja (2)

dipsas (6)

ophidion (4)

odryas (6)

agkistrodon (2)

heterodon (2)

bungarus (2)

pisthodon (2)

atheris (3)

bothro (6)
aspis (5)

Genus names used that did not match any of the above:

Bitis

Naja

Daboia

Ophiophagus

Ptyas

Lycodon

Dasypeltis

Bungarus

Dispholidus

Thelotornis

Crotalus

Agkistrodon

Sistrurus

Generic words used:

adder

amarilla

bandy

bardick

bécquer

bejuquilla

boomslang

bronzeback

cantil

cascabel

cascavel

coachwhip

cobra

copperhead

cottonmouth

couleuvre

cribo

crotale

cuatronarices

culebra

dhaman

diamondback

dugite

egg-eater

ferdelance

habu

hognose

jararaca

keelback

krait

kukri

lancehead

mamba

mamushi

massasauga

moccasin

mulga

myall

rattler

ringhals

rinkhals

schlange

serpente

serpiente

shieldtail

shovel-snout

sidewinder

sipo

skaapsteker

slug-eater

snail-eater

snake

taipan

terciopelo

urutu

vibora

yamakagashi

Not used (mostly because of too much irrelevant content):

asp
anaconda
bushmaster
racer
woma

Appendix 3: Species names used in GARD and those combined with them for spatial analysis.

| species name in GARD | species with no GARD map that were split from a GARD species, which we recombined for the map analysis |
| --- | --- |
| *Acanthophis rugosus* | *Acanthophis cryptamydros* |
| *Adelphicos quadrivirgatum* | *Adelphicos newmanorum, Adelphicos sargii, Adelphicos visoninum* |
| *Agkistrodon contortrix* | *Agkistrodon laticinctus* |
| *Agkistrodon piscivorus* | *Agkistrodon conanti* |
| *Ahaetulla nasuta* | *Ahaetulla isabellina, Ahaetulla oxyrhyncha* |
| *Ahaetulla pulverulenta* | *Ahaetulla anomala* |
| *Antaioserpens warro* | *Antaioserpens albiceps* |
| *Apostolepis assimilis* | *Apostolepis tertulianobeui* |
| *Apostolepis nigroterminata* | *Apostolepis borellii* |
| *Arizona elegans* | *Arizona pacata* |
| *Boa constrictor* | *Boa imperator, Boa nebulosa, Boa orophias* |
| *Boaedon fuliginosus* | *Boaedon angolensis, Boaedon perisilvestris, Boaedon variegatus* |
| *Borikenophis portoricensis* | *Borikenophis prymnus* |
| *Bothriechis nigroviridis* | *Bothriechis nubestris* |
| *Brachyurophis semifasciatus* | *Brachyurophis campbelli* |
| *Bungarus sindanus* | *Bungarus walli* |
| *Calliophis intestinalis* | *Calliophis bilineata, Calliophis nigrotaeniatus, Calliophis philippina, Calliophis suluensis* |
| *Cemophora coccinea* | *Cemophora lineri* |
| *Chilabothrus chrysogaster* | *Chilabothrus schwartzi* |
| *Chilabothrus monensis* | *Chilabothrus granti* |
| *Coelognathus erythrurus* | *Coelognathus philippinus* |
| *Coniophanes piceivittis* | *Coniophanes taeniata, Coniophanes taylori* |
| *Crotalus durissus* | *Crotalus unicolor, Crotalus vegrandis* |
| *Crotalus lepidus* | *Crotalus morulus* |
| *Crotalus mitchellii* | *Crotalus angelensis, Crotalus polisi, Crotalus pyrrhus, Crotalus thalassoporus* |
| *Crotalus molossus* | *Crotalus estebanensis, Crotalus ornatus* |
| *Crotalus oreganus* | *Crotalus concolor, Crotalus helleri, Crotalus lutosus* |
| *Crotalus ruber* | *Crotalus lorenzoensis* |
| *Cylindrophis ruffus* | *Cylindrophis burmanus* |
| *Dasypeltis scabra* | *Dasypeltis bazi, Dasypeltis crucifera, Dasypeltis taylori* |
| *Dendrelaphis caudolineolatus* | *Dendrelaphis effrenis* |
| *Dendrelaphis punctulatus* | *Dendrelaphis macrops, Dendrelaphis striolatus* |
| *Dipsadoboa shrevei* | *Dipsadoboa kageleri* |
| *Dipsas bucephala* | *Dipsas cisticeps* |
| *Dipsas peruana* | *Dipsas latifrontalis, Dipsas palmeri* |
| *Echis ocellatus* | *Echis romani* |
| *Eirenis persicus* | *Eirenis nigrofasciatus* |
| *Epictia goudotii* | *Epictia bakewelli, Epictia fallax* |
| *Epictia signata* | *Epictia amazonica* |
| *Epictia tenella* | *Epictia albifrons* |
| *Erythrolamprus reginae* | *Erythrolamprus macrosomus, Erythrolamprus oligolepis, Erythrolamprus zweifeli* |
| *Gloydius halys* | *Gloydius caraganus, Gloydius caucasicus, Gloydius cognatus, Gloydius liupanensis, Gloydius stejnegeri* |
| *Gloydius strauchi* | *Gloydius qinlingensis* |
| *Hemibungarus calligaster* | *Hemibungarus gemianulis, Hemibungarus mcclungi* |
| *Indotyphlops braminus* | *Indotyphlops fletcheri* |
| *Lampropeltis getula* | *Lampropeltis catalinensis* |
| *Lampropeltis mexicana* | *Lampropeltis greeri, Lampropeltis leonis* |
| *Lampropeltis pyromelana* | *Lampropeltis knoblochi* |
| *Leptodeira annulata* | *Leptodeira ashmeadii, Leptodeira rhombifera* |
| *Leptodeira septentrionalis* | *Leptodeira polysticta* |
| *Lichanura trivirgata* | *Lichanura orcutti* |
| *Lycodon subcinctus* | *Lycodon sealei* |
| *Mastigodryas melanolomus* | *Mastigodryas alternatus* |
| *Melanophidium punctatum* | *Melanophidium khairei* |
| *Micrurus hemprichii* | *Micrurus boicora* |
| *Micrurus lemniscatus* | *Micrurus diutius* |
| *Micrurus ornatissimus* | *Micrurus tikuna* |
| *Micrurus pyrrhocryptus* | *Micrurus tricolor* |
| *Morelia spilota* | *Morelia imbricata* |
| *Morelia viridis* | *Morelia azurea* |
| *Naja melanoleuca* | *Naja guineensis, Naja peroescobari, Naja savannula, Naja subfulva* |
| *Natrix natrix* | *Natrix astreptophora, Natrix helvetica* |
| *Ninia atrata* | *Ninia teresitae* |
| *Oligodon waandersi* | *Oligodon propinquus* |
| *Ophryacus undulatus* | *Ophryacus smaragdinus* |
| *Oxybelis aeneus* | *Oxybelis microphthalmus, Oxybelis potosiensis, Oxybelis vittatus* |
| *Pareas formosensis* | *Pareas komaii* |
| *Pareas margaritophorus* | *Pareas andersonii, Pareas macularius, Pareas modestus* |
| *Philodryas mattogrossensis* | *Philodryas erlandi* |
| *Philothamnus natalensis* | *Philothamnus occidentalis* |
| *Phrynonax poecilonotus* | *Phrynonax polylepis* |
| *Pituophis vertebralis* | *Pituophis insulanus* |
| *Platyceps karelini* | *Platyceps rogersi* |
| *Platyceps najadum* | *Platyceps atayevi, Platyceps schmidtleri* |
| *Platyceps rhodorachis* | *Platyceps rhodorachis, Platyceps saharicus* |
| *Polemon fulvicollis* | *Polemon graueri* |
| *Psammophylax rhombeatus* | *Psammophylax ocellatus* |
| *Psammophylax variabilis* | *Psammophylax multisquamis* |
| *Pseudorabdion longiceps* | *Pseudorabdion torquatum* |
| *Rena humilis* | *Rena dugesii, Rena segrega* |
| *Rena myopica* | *Rena iversoni* |
| *Rhabdophis nuchalis* | *Rhabdophis pentasupralabialis* |
| *Rhinocheilus lecontei* | *Rhinocheilus antonii, Rhinocheilus etheridgei* |
| *Rhynchocalamus melanocephalus* | *Rhynchocalamus satunini* |
| *Salvadora hexalepis* | *Salvadora deserticola* |
| *Sinomicrurus macclellandi* | *Sinomicrurus nigriventer* |
| *Sistrurus catenatus* | *Sistrurus tergeminus* |
| *Sonora semiannulata* | *Sonora episcopa, Sonora mosaueri, Sonora taylori* |
| *Stegonotus cucullatus* | *Stegonotus australis, Stegonotus keyensis, Stegonotus reticulatus* |
| *Stegonotus florensis* | *Stegonotus sutteri* |
| *Stegonotus modestus* | *Stegonotus aruensis, Stegonotus lividus* |
| *Storeria dekayi* | *Storeria victa* |
| *Synophis bicolor* | *Synophis bogerti* |
| *Telescopus dhara* | *Telescopus obtusus* |
| *Thamnophis elegans* | *Thamnophis errans* |
| *Thamnophis rufipunctatus* | *Thamnophis unilabialis* |
| *Trilepida macrolepis* | *Trilepida affinis* |
| *Vipera berus* | *Vipera nikolskii, Vipera walser* |
| *Vipera ursinii* | *Vipera graeca* |
| *Xerotyphlops vermicularis* | *Xerotyphlops syriacus* |

| species names in GARD that were mapped separately but were combined into a single map for analysis because of synonymy since 2017 | currently valid species (Reptile Database Dec 2020) |
| --- | --- |
| *Atractus balzani, Atractus boettgeri, Atractus paravertebralis, Atractus taeniatus* | *Atractus emmeli* |
| *Trimeresurus barati, Trimeresurus buniana, Trimeresurus fucatus, Trimeresurus toba* | *Trimeresurus sabahi* |
| *Bothrochilus biakensis, Bothrochilus huonensis* | *Leiopython albertisii* |
| *Bothrochilus meridionalis, Bothrochilus montanus* | *Leiopython fredparkeri* |
| *Vipera magnifica, Vipera pontica* | *Vipera kaznakovi* |

Appendix 4: 781 species from December 2020 Reptile Database release with no photos in our dataset; see online supplement for table sortable by family, continent, authority, and medical importance

*Achalinus ater*

*Achalinus hainanus*

*Achalinus jinggangensis*

*Achalinus zugorum*

*Acutotyphlops banaorum*

*Acutotyphlops infralabialis*

*Acutotyphlops kunuaensis*

*Adelophis foxi*

*Adelphicos ibarrorum*

*Aeluroglena cucullata*

*Afrotyphlops anomalus*

*Afrotyphlops blanfordii*

*Afrotyphlops brevis*

*Afrotyphlops calabresii*

*Afrotyphlops cuneirostris*

*Afrotyphlops gierrai*

*Afrotyphlops kaimosae*

*Afrotyphlops liberiensis*

*Afrotyphlops nanus*

*Afrotyphlops platyrhynchus*

*Afrotyphlops rondoensis*

*Afrotyphlops steinhausi*

*Afrotyphlops tanganicanus*

*Afrotyphlops usambaricus*

*Aipysurus fuscus*

*Aipysurus pooleorum*

*Aipysurus tenuis*

*Amblyodipsas dimidiata*

*Amblyodipsas rodhaini*

*Amblyodipsas teitana*

*Amerotyphlops lehneri*

*Amerotyphlops yonenagae*

*Amnesteophis melanauchen*

*Anilios batillus*

*Anilios fossor*

*Anilios insperatus*

*Anilios leucoproctus*

*Anilios longissimus*

*Anilios margaretae*

*Anilios micromma*

*Anilios minimus*

*Anilios robertsi*

*Anilios systenos*

*Anilios tovelli*

*Anilios troglodytes*

*Anilios vagurima*

*Anilios yampiensis*

*Anilios yirrikalae*

*Anomalepis aspinosus*

*Anomalepis flavapices*

*Anoplohydrus aemulans*

*Antillotyphlops annae*

*Antillotyphlops catapontus*

*Antillotyphlops granti*

*Antillotyphlops guadeloupensis*

*Antillotyphlops monastus*

*Antillotyphlops monensis*

*Antillotyphlops naugus*

*Antillotyphlops richardi*

*Aparallactus moeruensis*

*Aparallactus niger*

*Apostolepis breviceps*

*Apostolepis cerradoensis*

*Apostolepis dorbignyi*

*Apostolepis goiasensis*

*Apostolepis intermedia*

*Apostolepis lineata*

*Apostolepis nigroterminata*

*Apostolepis quirogai*

*Apostolepis serrana*

*Apostolepis striata*

*Apostolepis underwoodi*

*Apostolepis vittata*

*Aprosdoketophis andreonei*

*Argyrogena vittacaudata*

*Argyrophis bothriorhynchus*

*Argyrophis fuscus*

*Argyrophis giadinhensis*

*Argyrophis hypsobothrius*

*Argyrophis klemmeri*

*Argyrophis koshunensis*

*Argyrophis roxaneae*

*Argyrophis siamensis*

*Argyrophis trangensis*

*Arrhyton ainictum*

*Arrhyton tanyplectum*

*Aspidura deraniyagalae*

*Atheris hetfieldi*

*Atheris katangensis*

*Atractaspis battersbyi*

*Atractaspis boulengeri*

*Atractaspis engdahli*

*Atractaspis leucomelas*

*Atractaspis magrettii*

*Atractaspis micropholis*

*Atractaspis phillipsi*

*Atractaspis scorteccii*

*Atractus aboiporu*

*Atractus acheronius*

*Atractus alphonsehogei*

*Atractus altagratiae*

*Atractus alytogrammus*

*Atractus andinus*

*Atractus apophis*

*Atractus atratus*

*Atractus attenuates*

*Atractus avernus*

*Atractus ayeush*

*Atractus boulengerii*

*Atractus careolepis*

*Atractus chthonius*

*Atractus depressiocellus*

*Atractus duidensis*

*Atractus echidna*

*Atractus ecuadorensis*

*Atractus edioi*

*Atractus esepe*

*Atractus heliobelluomini*

*Atractus heyeri*

*Atractus hoogmoedi*

*Atractus hostilitractus*

*Atractus insipidus*

*Atractus loveridgei*

*Atractus macondo*

*Atractus mariselae*

*Atractus matthewi*

*Atractus medusa*

*Atractus meridensis*

*Atractus micheleae*

*Atractus mijaresi*

*Atractus multidentatus*

*Atractus nasutus*

*Atractus nigricaudus*

*Atractus nigriventris*

*Atractus ochrosetrus*

*Atractus oculotemporalis*

*Atractus pauciscutatus*

*Atractus peruvianus*

*Atractus punctiventris*

*Atractus steyermarki*

*Atractus stygius*

*Atractus surucucu*

*Atractus tamaensis*

*Atractus taphorni*

*Atractus thalesdelemai*

*Atractus trivittatus*

*Atractus variegatus*

*Atractus ventrimaculatus*

*Atractus vertebrolineatus*

*Atractus vittatus*

*Boaedon littoralis*

*Boaedon maculatus*

*Boaedon upembae*

*Boiga saengsomi*

*Borikenophis sanctaecrucis*

*Borikenophis variegatus*

*Brachyorrhos albus*

*Brachyorrhos gastrotaenius*

*Brachyorrhos wallacei*

*Brygophis coulangesi*

*Buhoma procterae*

*Bungarus magnimaculatus*

*Calamaria abstrusa*

*Calamaria acutirostris*

*Calamaria alidae*

*Calamaria apraeocularis*

*Calamaria boesemani*

*Calamaria buchi*

*Calamaria ceramensis*

*Calamaria concolor*

*Calamaria crassa*

*Calamaria curta*

*Calamaria doederleini*

*Calamaria eiselti*

*Calamaria everetti*

*Calamaria forcarti*

*Calamaria gracillima*

*Calamaria javanica*

*Calamaria joloensis*

*Calamaria lateralis*

*Calamaria lautensis*

*Calamaria leucogaster*

*Calamaria lumholtzi*

*Calamaria mecheli*

*Calamaria melanota*

*Calamaria muelleri*

*Calamaria palavanensis*

*Calamaria pfefferi*

*Calamaria prakkei*

*Calamaria rebentischi*

*Calamaria sangi*

*Calamaria suluensis*

*Calamaria sumatrana*

*Calamaria thanhi*

*Calamaria ulmeri*

*Calamodontophis ronaldoi*

*Calamophis jobiensis*

*Calamophis katesandersae*

*Calamophis ruuddelangi*

*Calamophis sharonbrooksae*

*Calamorhabdium kuekenthali*

*Calliophis salitan*

*Cathetorhinus melanocephalus*

*Cenaspis aenigma*

*Cerberus microlepis*

*Chamaelycus christyi*

*Chamaelycus parkeri*

*Chersodromus australis*

*Chilorhinophis butleri*

*Chironius leucometapus*

*Chironius vincenti*

*Clelia errabunda*

*Clelia langeri*

*Compsophis vinckei*

*Compsophis zeny*

*Coniophanes andresensis*

*Coniophanes joanae*

*Coniophanes longinquus*

*Conophis morai*

*Conopsis amphisticha*

*Coronelaps lepidus*

*Crotaphopeltis braestrupi*

*Cryptophis incredibilis*

*Cubatyphlops anchaurus*

*Cubatyphlops caymanensis*

*Cubatyphlops epactius*

*Cubatyphlops golyathi*

*Cubatyphlops paradoxus*

*Cubatyphlops satelles*

*Cubophis brooksi*

*Cubophis fuscicauda*

*Cubophis ruttyi*

*Cylindrophis aruensis*

*Cylindrophis boulengeri*

*Cylindrophis isolepis*

*Cylindrophis osheai*

*Cylindrophis slowinskii*

*Dasypeltis arabica*

*Dendrelaphis flavescens*

*Dendrelaphis gastrostictus*

*Dendrelaphis grismeri*

*Dendrelaphis hollinrakei*

*Dendrelaphis keiensis*

*Dendrelaphis lorentzii*

*Dendrelaphis modestus*

*Dendrelaphis oliveri*

*Dendrelaphis papuensis*

*Dendrelaphis walli*

*Diaphorolepis laevis*

*Dipsas baliomelas*

*Dipsas chaparensis*

*Dipsas maxillaris*

*Dipsas oligozonata*

*Dipsas pakaraima*

*Dipsas schunkii*

*Dipsas vagus*

*Drymarchon margaritae*

*Drymoluber apurimacensis*

*Echis megalocephalus*

*Eirenis aurolineatus*

*Eirenis rafsanjanicus*

*Eirenis rechingeri*

*Elapoidis sumatrana*

*Elapsoidea broadleyi*

*Elapsoidea laticincta*

*Emmochliophis fugleri*

*Emmochliophis miops*

*Enhydris innominata*

*Epacrophis boulengeri*

*Epacrophis drewesi*

*Epacrophis reticulatus*

*Epictia alfredschmidti*

*Epictia melanura*

*Epictia peruviana*

*Epictia rioignis*

*Epictia rubrolineata*

*Epictia undecimstriata*

*Epictia unicolor*

*Epictia vellardi*

*Epictia wynni*

*Erythrolamprus albertguentheri*

*Erythrolamprus andinus*

*Erythrolamprus guentheri*

*Erythrolamprus ingeri*

*Erythrolamprus janaleeae*

*Erythrolamprus perfuscus*

*Erythrolamprus pyburni*

*Erythrolamprus rochai*

*Erythrolamprus subocularis*

*Erythrolamprus torrenicola*

*Erythrolamprus trebbaui*

*Erythrolamprus williamsi*

*Eryx somalicus*

*Eryx vittatus*

*Etheridgeum pulchrum*

*Euprepiophis perlaceus*

*Ficimia ramirezi*

*Ficimia ruspator*

*Ficimia variegata*

*Geophis isthmicus*

*Geophis juarezi*

*Geophis juliai*

*Geophis laticollaris*

*Geophis rostralis*

*Gerrhopilus addisoni*

*Gerrhopilus andamanensis*

*Gerrhopilus ater*

*Gerrhopilus bisubocularis*

*Gerrhopilus depressiceps*

*Gerrhopilus eurydice*

*Gerrhopilus floweri*

*Gerrhopilus fredparkeri*

*Gerrhopilus hades*

*Gerrhopilus hedraeus*

*Gerrhopilus inornatus*

*Gerrhopilus lestes*

*Gerrhopilus mcdowelli*

*Gerrhopilus oligolepis*

*Gerrhopilus tindalli*

*Gongylosoma nicobariensis*

*Gonionotophis grantii*

*Gyiophis maculosa*

*Hebius arquus*

*Hebius celebicum*

*Hebius chapaensis*

*Hebius clerki*

*Hebius concelarum*

*Hebius frenatum*

*Hebius groundwateri*

*Hebius ishigakiense*

*Hebius johannis*

*Hebius kerinciense*

*Hebius lacrima*

*Hebius modestum*

*Hebius nicobariense*

*Hebius parallelum*

*Hebius sarasinorum*

*Hebius taronense*

*Helicops tapajonicus*

*Helicops yacu*

*Helminthophis flavoterminatus*

*Herpetoreas burbrinki*

*Herpetoreas pealii*

*Heurnia ventromaculata*

*Homalopsis hardwickii*

*Hydrablabes praefrontalis*

*Hydraethiops laevis*

*Hydromorphus dunni*

*Hydrophis atriceps*

*Hydrophis bituberculatus*

*Hydrophis cantoris*

*Hydrophis hendersoni*

*Hydrophis klossi*

*Hydrophis macdowelli*

*Hydrophis melanosoma*

*Hydrophis nigrocinctus*

*Hydrophis obscurus*

*Hydrophis pacificus*

*Hydrophis parviceps*

*Hydrophis stricticollis*

*Hydrophis torquatus*

*Hydrophis vorisi*

*Hypoptophis wilsonii*

*Hypsiglena unaocularus*

*Hypsirhynchus ater*

*Hypsirhynchus funereus*

*Hypsirhynchus melanichnus*

*Hypsirhynchus scalaris*

*Hypsiscopus matannensis*

*Ialtris agyrtes*

*Ialtris dorsalis*

*Ialtris haetianus*

*Ialtris parishi*

*Iguanognathus werneri*

*Imantodes guane*

*Indotyphlops ahsanai*

*Indotyphlops exiguus*

*Indotyphlops filiformis*

*Indotyphlops jerdoni*

*Indotyphlops lankaensis*

*Indotyphlops lazelli*

*Indotyphlops leucomelas*

*Indotyphlops longissimus*

*Indotyphlops loveridgei*

*Indotyphlops madgemintonae*

*Indotyphlops malcolmi*

*Indotyphlops meszoelyi*

*Indotyphlops mollyozakiae*

*Indotyphlops pammeces*

*Indotyphlops schmutzi*

*Indotyphlops tenebrarum*

*Indotyphlops tenuicollis*

*Indotyphlops veddae*

*Indotyphlops violaceus*

*Ithycyphus goudoti*

*Karnsophis siantaris*

*Kladirostratus togoensis*

*Kualatahan pahangensis*

*Leptotyphlops emini*

*Leptotyphlops howelli*

*Leptotyphlops keniensis*

*Leptotyphlops latirostris*

*Leptotyphlops mbanjensis*

*Leptotyphlops nigroterminus*

*Leptotyphlops pembae*

*Leptotyphlops pungwensis*

*Letheobia acutirostrata*

*Letheobia angeli*

*Letheobia caeca*

*Letheobia coecatus*

*Letheobia crossii*

*Letheobia debilis*

*Letheobia erythraea*

*Letheobia feae*

*Letheobia jubana*

*Letheobia kibarae*

*Letheobia largeni*

*Letheobia leucosticta*

*Letheobia lumbriciformis*

*Letheobia manni*

*Letheobia newtoni*

*Letheobia pallida*

*Letheobia pauwelsi*

*Letheobia pembana*

*Letheobia praeocularis*

*Letheobia rufescens*

*Letheobia stejnegeri*

*Letheobia sudanensis*

*Letheobia swahilica*

*Letheobia toritensis*

*Letheobia uluguruensis*

*Letheobia wittei*

*Letheobia zenkeri*

*Limaformosa savorgnani*

*Lioheterophis iheringi*

*Liopholidophis baderi*

*Liotyphlops argaleus*

*Liotyphlops haadi*

*Liotyphlops schubarti*

*Lycodon alcalai*

*Lycodon bibonius*

*Lycodon chrysoprateros*

*Lycodon fausti*

*Lycodon kundui*

*Lycodon multizonatus*

*Lycodon philippinus*

*Lycodon solivagus*

*Lycodon stormi*

*Lycodon tessellatus*

*Lycodon zayuensis*

*Lycodonomorphus leleupi*

*Lycodonomorphus subtaeniatus*

*Lycodryas carleti*

*Lycodryas guentheri*

*Lycodryas inornatus*

*Lycophidion hellmichi*

*Lycophidion irroratum*

*Lycophidion meleagre*

*Lycophidion pembanum*

*Lycophidion semiannule*

*Lytorhynchus gasperetti*

*Macrocalamus jasoni*

*Macrocalamus vogeli*

*Madatyphlops albanalis*

*Madatyphlops boettgeri*

*Madatyphlops cariei*

*Madatyphlops comorensis*

*Madatyphlops madagascariensis*

*Madatyphlops ocularis*

*Madatyphlops reuteri*

*Malayotyphlops andyi*

*Malayotyphlops canlaonensis*

*Malayotyphlops collaris*

*Malayotyphlops denrorum*

*Malayotyphlops hypogius*

*Malayotyphlops koekkoeki*

*Malayotyphlops kraalii*

*Malayotyphlops luzonensis*

*Malayotyphlops manilae*

*Malayotyphlops ruber*

*Mastigodryas amarali*

*Mehelya egbensis*

*Mehelya gabouensis*

*Mehelya laurenti*

*Meizodon krameri*

*Meizodon plumbiceps*

*Micrurus bogerti*

*Micrurus diana*

*Micrurus margaritiferus*

*Micrurus meridensis*

*Micrurus nattereri*

*Micrurus pacaraimae*

*Micrurus petersi*

*Micrurus spurrelli*

*Micrurus stuarti*

*Mintonophis pakistanicus*

*Mitophis asbolepis*

*Mitophis calypso*

*Mitophis leptepileptus*

*Mitophis pyrites*

*Myersophis alpestris*

*Myriopholis boueti*

*Myriopholis burii*

*Myriopholis cairi*

*Myriopholis erythraeus*

*Myriopholis ionidesi*

*Myriopholis lanzai*

*Myriopholis occipitalis*

*Myriopholis parkeri*

*Myriopholis perreti*

*Myriopholis tanae*

*Myriopholis yemenica*

*Naja christyi*

*Namibiana latifrons*

*Natriciteres bipostocularis*

*Natriciteres pembana*

*Oligodon annamensis*

*Oligodon cruentatus*

*Oligodon eberhardti*

*Oligodon erythrorhachis*

*Oligodon hamptoni*

*Oligodon jintakunei*

*Oligodon joynsoni*

*Oligodon lipipengi*

*Oligodon lungshenensis*

*Oligodon maculatus*

*Oligodon mcdougalli*

*Oligodon melaneus*

*Oligodon melanozonatus*

*Oligodon meyerinkii*

*Oligodon modestus*

*Oligodon moricei*

*Oligodon nikhili*

*Oligodon ocellatus*

*Oligodon petronellae*

*Oligodon planiceps*

*Oligodon praefrontalis*

*Oligodon pulcherrimus*

*Oligodon saintgironsi*

*Oligodon torquatus*

*Oligodon travancoricus*

*Oligodon unicolor*

*Oligodon vertebralis*

*Oligodon wagneri*

*Omoadiphas cannula*

*Omoadiphas texiguatensis*

*Ophryacus sphenophrys*

*Opisthotropis alcalai*

*Opisthotropis atra*

*Opisthotropis daovantieni*

*Opisthotropis guangxiensis*

*Opisthotropis jacobi*

*Opisthotropis kikuzatoi*

*Opisthotropis rugosa*

*Opisthotropis spenceri*

*Opisthotropis tamdaoensis*

*Oxyrhopus doliatus*

*Oxyrhopus marcapatae*

*Parafimbrios vietnamensis*

*Parahydrophis mertoni*

*Parapistocalamus hedigeri*

*Pareas mengziensis*

*Pareas vindumi*

*Phalotris concolor*

*Phalotris nigrilatus*

*Philodryas boliviana*

*Philodryas cordata*

*Philothamnus girardi*

*Philothamnus hughesi*

*Philothamnus pobeguini*

*Philothamnus ruandae*

*Phisalixella iarakaensis*

*Platyceps afarensis*

*Platyceps insulanus*

*Platyceps largeni*

*Platyceps noeli*

*Platyceps sindhensis*

*Platyceps somalicus*

*Platyceps variabilis*

*Plectrurus aureus*

*Poecilopholis cameronensis*

*Polemon christyi*

*Polemon gabonensis*

*Polemon griseiceps*

*Polemon robustus*

*Prosymna ornatissima*

*Prosymna semifasciata*

*Prosymna somalica*

*Psammophis ansorgii*

*Pseudoboa haasi*

*Pseudoboodon gascae*

*Pseudorabdion ater*

*Pseudorabdion eiselti*

*Pseudorabdion modiglianii*

*Pseudorabdion montanum*

*Pseudorabdion oxycephalum*

*Pseudorabdion sarasinorum*

*Pseudorabdion saravacense*

*Pseudorabdion sirambense*

*Pseudorabdion talonuran*

*Pseudorabdion taylori*

*Pseudoxyrhopus ankafinaensis*

*Ramphotyphlops becki*

*Ramphotyphlops cumingii*

*Ramphotyphlops depressus*

*Ramphotyphlops lorenzi*

*Ramphotyphlops mansuetus*

*Ramphotyphlops marxi*

*Ramphotyphlops similis*

*Ramphotyphlops suluensis*

*Ramphotyphlops supranasalis*

*Ramphotyphlops willeyi*

*Rena bressoni*

*Rhabdophis akraios*

*Rhabdophis callichroma*

*Rhabdophis callistus*

*Rhadinaea myersi*

*Rhadinaea omiltemana*

*Rhadinaea quinquelineata*

*Rhadinaea sargenti*

*Rhadinaea vermiculaticeps*

*Rhadinella donaji*

*Rhadinella hannsteini*

*Rhadinella posadasi*

*Rhadinella stadelmani*

*Rhadinella tolpanorum*

*Rhadinophanes monticola*

*Rhinoguinea magna*

*Rhinoleptus koniagui*

*Rhinophis dorsimaculatus*

*Rhinophis mendisi*

*Rhinophis porrectus*

*Rhinophis travancoricus*

*Rhinophis tricoloratus*

*Rhinotyphlops ataeniatus*

*Rhinotyphlops leucocephalus*

*Rhinotyphlops scortecci*

*Saphenophis antioquiensis*

*Saphenophis atahuallpae*

*Saphenophis sneiderni*

*Saphenophis tristriatus*

*Scaphiophis raffreyi*

*Sibon dunni*

*Sibon linearis*

*Sibynophis bistrigatus*

*Sibynophis bivittatus*

*Smithophis linearis*

*Spalerosophis josephscorteccii*

*Stegonotus ayamaru*

*Stegonotus melanolabiatus*

*Stegonotus poechi*

*Stoliczkia khasiensis*

*Synophis plectovertebralis*

*Tachymenis affinis*

*Tachymenis attenuata*

*Tachymenis tarmensis*

*Tantilla albiceps*

*Tantilla bairdi*

*Tantilla briggsi*

*Tantilla cascadae*

*Tantilla coronadoi*

*Tantilla jani*

*Tantilla johnsoni*

*Tantilla nigra*

*Tantilla oaxacae*

*Tantilla olympia*

*Tantilla robusta*

*Tantilla shawi*

*Tantilla slavensi*

*Tantilla tayrae*

*Tantilla tecta*

*Tantilla triseriata*

*Tantilla vulcani*

*Telescopus gezirae*

*Tetralepis fruhstorferi*

*Thamnodynastes ceibae*

*Thamnodynastes chimanta*

*Thamnodynastes corocoroensis*

*Thamnodynastes duida*

*Thamnodynastes marahuaquensis*

*Thrasops schmidti*

*Toxicocalamus grandis*

*Toxicocalamus holopelturus*

*Toxicocalamus longissimus*

*Toxicocalamus misimae*

*Toxicocalamus pachysomus*

*Toxicocalamus spilolepidotus*

*Toxicocalamus stanleyanus*

*Trachischium laeve*

*Tretanorhinus mocquardi*

*Tretanorhinus taeniatus*

*Tricheilostoma broadleyi*

*Tricheilostoma dissimilis*

*Tricheilostoma sundewalli*

*Trilepida anthracina*

*Trilepida brevissima*

*Trilepida fuliginosa*

*Trilepida nicefori*

*Trimeresurus tibetanus*

*Trimetopon gracile*

*Trimetopon simile*

*Trimetopon viquezi*

*Tropidonophis aenigmaticus*

*Tropidonophis dahlii*

*Tropidonophis doriae*

*Tropidonophis elongatus*

*Tropidonophis halmahericus*

*Tropidonophis hypomelas*

*Tropidonophis mcdowelli*

*Tropidonophis novaeguineae*

*Tropidonophis parkeri*

*Tropidonophis picturatus*

*Tropidonophis punctiventris*

*Tropidonophis statisticus*

*Tropidophis battersbyi*

*Tropidophis bucculentus*

*Tropidophis hardyi*

*Tropidophis jamaicensis*

*Tropidophis morenoi*

*Tropidophis nigriventris*

*Tropidophis parkeri*

*Tropidophis schwartzi*

*Tropidophis stejnegeri*

*Tropidophis stullae*

*Typhlops agoralionis*

*Typhlops capitulatus*

*Typhlops gonavensis*

*Typhlops leptolepis*

*Typhlops oxyrhinus*

*Typhlops pachyrhinus*

*Typhlops proancylops*

*Typhlops silus*

*Typhlops sulcatus*

*Typhlops sylleptor*

*Typhlops syntherus*

*Uropeltis beddomii*

*Uropeltis broughami*

*Uropeltis macrorhyncha*

*Uropeltis maculata*

*Uropeltis nitida*

*Uropeltis rubrolineata*

*Urotheca dumerilli*

*Urotheca myersi*

*Vermicella multifasciata*

*Xenocalamus michelli*

*Xenochrophis bellulus*

*Xenophidion acanthognathus*

*Xerotyphlops etheridgei*

*Xerotyphlops luristanicus*

Appendix 5: Parameter estimates from top model. Intercept corresponds to non-venomous non-blindsnakes from Africa. Range size is in millions of km^2^, population density is in thousands of people per km^2^.

| term | estimate | standard error | statistic | p value | 95% confidence interval lower bound | 95% confidence interval upper bound |
| --- | --- | --- | --- | --- | --- | --- |
| Intercept | 3.052061 | 0.112437 | 27.144563 | 8.035E-147 | 2.831608 | 3.272514 |
| ln(range size) | 1.141521 | 0.050562 | 22.576723 | 3.612E-105 | 1.042385 | 1.240656 |
| ln(population density) | 3.343764 | 0.405954 | 8.236806 | 2.508E-16 | 2.547821 | 4.139708 |
| ln(population density^2^) | -2.077795 | 0.364858 | -5.694799 | 1.342E-08 | -2.793162 | -1.362427 |
| Asia | -0.168335 | 0.079734 | -2.111193 | 3.483E-02 | -0.324668 | -0.012002 |
| Australia | 0.797957 | 0.115377 | 6.916067 | 5.539E-12 | 0.571740 | 1.024174 |
| Europe | 1.592478 | 0.248072 | 6.419406 | 1.561E-10 | 1.106089 | 2.078867 |
| USA + Canada | 2.963125 | 0.131127 | 22.597431 | 2.405E-105 | 2.706029 | 3.220222 |
| Latin America | 0.238928 | 0.072621 | 3.290052 | 1.012E-03 | 0.096541 | 0.381315 |
| medically-important | 0.955655 | 0.072629 | 13.158107 | 1.367E-38 | 0.813254 | 1.098056 |
| blindsnake | -0.965937 | 0.081997 | -11.780120 | 2.046E-31 | -1.126707 | -0.805167 |
| year since 1758 | -0.009659 | 0.000491 | -19.684392 | 1.040E-81 | -0.010621 | -0.008697 |

Appendix 6: Full model selection table. NA means the term was not in the model. The top model was the global model. Intercept corresponds to non-venomous non-blindsnakes from Africa. Range size is in millions of km^2^, population density is in thousands of people per km^2^. Response variable is ln(number of photos).

| (Intercept) | blindsnake | global region | ln(pop_dens) | ln(pop_dens^2^) | ln(range size) | mivs | years1758 | df | logLik | AIC_c_ | delta | weight |
| --- | --- | --- | --- | --- | --- | --- | --- | --- | --- | --- | --- | --- |
| 3.05206 | + | + | 3.34376 | -2.07779 | 1.14152 | + | -0.00966 | 13 | -6056.25 | 12139 | 0 | 1.00 |
| 3.20387 | + | + | 1.41548 | NA | 1.12095 | + | -0.01014 | 12 | -6072.44 | 12169 | 30 | 0.00 |
| 3.36635 | + | + | NA | 0.42889 | 1.08122 | + | -0.01072 | 12 | -6089.95 | 12204 | 65 | 0.00 |
| 3.38632 | + | + | NA | NA | 1.07158 | + | -0.01081 | 11 | -6092.19 | 12206 | 68 | 0.00 |
| 2.89390 | NA | + | 3.24560 | -2.02940 | 1.17859 | + | -0.01012 | 12 | -6124.48 | 12273 | 134 | 0.00 |
| 3.04304 | NA | + | 1.36250 | NA | 1.15829 | + | -0.01059 | 11 | -6139.32 | 12301 | 162 | 0.00 |
| 3.18259 | + | + | 3.39169 | -2.12839 | 1.16891 | NA | -0.00950 | 12 | -6140.96 | 12306 | 167 | 0.00 |
| 3.20129 | NA | + | NA | 0.40404 | 1.11951 | + | -0.01114 | 11 | -6155.01 | 12332 | 193 | 0.00 |
| 3.22041 | NA | + | NA | NA | 1.11036 | + | -0.01123 | 10 | -6156.92 | 12334 | 195 | 0.00 |
| 3.33873 | + | + | 1.41645 | NA | 1.14796 | NA | -0.01000 | 11 | -6157.11 | 12336 | 198 | 0.00 |
| 3.50215 | + | + | NA | 0.41414 | 1.10789 | NA | -0.01058 | 11 | -6173.94 | 12370 | 231 | 0.00 |
| 3.52132 | + | + | NA | NA | 1.09857 | NA | -0.01066 | 10 | -6175.92 | 12372 | 233 | 0.00 |
| 3.01457 | NA | + | 3.28208 | -2.07871 | 1.21763 | NA | -0.01002 | 11 | -6230.95 | 12484 | 345 | 0.00 |
| 1.29831 | + | + | 5.44699 | -3.32145 | 1.66543 | + | NA | 12 | -6240.22 | 12505 | 366 | 0.00 |
| 3.16775 | NA | + | 1.35320 | NA | 1.19697 | NA | -0.01051 | 10 | -6245.56 | 12511 | 373 | 0.00 |
| 3.32582 | NA | + | NA | 0.38202 | 1.15801 | NA | -0.01106 | 10 | -6260.26 | 12541 | 402 | 0.00 |
| 3.34378 | NA | + | NA | NA | 1.14932 | NA | -0.01114 | 9 | -6261.86 | 12542 | 403 | 0.00 |
| 1.40370 | + | + | 2.44289 | NA | 1.67478 | + | NA | 11 | -6278.21 | 12579 | 440 | 0.00 |
| 4.59995 | + | + | 2.01667 | -1.48928 | NA | + | -0.01549 | 12 | -6294.31 | 12613 | 474 | 0.00 |
| 4.68923 | + | + | 0.64469 | NA | NA | + | -0.01576 | 11 | -6301.59 | 12625 | 487 | 0.00 |
| 4.74384 | + | + | NA | NA | NA | + | -0.01596 | 10 | -6305.27 | 12631 | 492 | 0.00 |
| 4.74319 | + | + | NA | 0.03576 | NA | + | -0.01596 | 11 | -6305.25 | 12633 | 494 | 0.00 |
| 1.45172 | + | + | 5.46014 | -3.35068 | 1.68360 | NA | NA | 11 | -6311.04 | 12644 | 506 | 0.00 |
| 1.02296 | NA | + | 5.44908 | -3.33369 | 1.73608 | + | NA | 11 | -6318.94 | 12660 | 521 | 0.00 |
| 1.51033 | + | + | NA | 0.82615 | 1.66087 | + | NA | 11 | -6325.04 | 12672 | 534 | 0.00 |
| 1.51955 | + | + | NA | NA | 1.65140 | + | NA | 10 | -6332.28 | 12685 | 546 | 0.00 |
| 1.55877 | + | + | 2.42953 | NA | 1.69312 | NA | NA | 10 | -6348.11 | 12716 | 578 | 0.00 |
| 1.12826 | NA | + | 2.43389 | NA | 1.74559 | + | NA | 10 | -6355.47 | 12731 | 592 | 0.00 |
| 3.81390 | + | NA | 1.48772 | -1.09190 | 1.02353 | + | -0.01199 | 8 | -6367.76 | 12752 | 613 | 0.00 |
| 3.90186 | + | NA | 0.52534 | NA | 1.01088 | + | -0.01224 | 7 | -6371.69 | 12757 | 619 | 0.00 |
| 4.47872 | NA | + | 1.85795 | -1.41344 | NA | + | -0.01622 | 11 | -6368.64 | 12759 | 621 | 0.00 |
| 3.99288 | + | NA | NA | NA | 0.98758 | + | -0.01252 | 6 | -6374.27 | 12761 | 622 | 0.00 |
| 3.98793 | + | NA | NA | 0.06856 | 0.98947 | + | -0.01250 | 7 | -6374.22 | 12762 | 624 | 0.00 |
| 4.56403 | NA | + | 0.55625 | NA | NA | + | -0.01647 | 10 | -6374.92 | 12770 | 631 | 0.00 |
| 4.61192 | NA | + | NA | NA | NA | + | -0.01664 | 9 | -6377.54 | 12773 | 635 | 0.00 |
| 4.61207 | NA | + | NA | -0.00796 | NA | + | -0.01664 | 10 | -6377.54 | 12775 | 637 | 0.00 |
| 4.77977 | + | + | 2.03392 | -1.52840 | NA | NA | -0.01547 | 11 | -6378.72 | 12780 | 641 | 0.00 |
| 4.87181 | + | + | 0.62586 | NA | NA | NA | -0.01575 | 10 | -6386.01 | 12792 | 653 | 0.00 |
| 4.92460 | + | + | NA | NA | NA | NA | -0.01594 | 9 | -6389.31 | 12797 | 658 | 0.00 |
| 4.92443 | + | + | NA | 0.00966 | NA | NA | -0.01594 | 10 | -6389.31 | 12799 | 660 | 0.00 |
| 1.66470 | + | + | NA | 0.80687 | 1.67909 | NA | NA | 10 | -6392.81 | 12806 | 667 | 0.00 |
| 1.67334 | + | + | NA | NA | 1.66978 | NA | NA | 9 | -6399.45 | 12817 | 678 | 0.00 |
| 1.23495 | NA | + | NA | 0.81549 | 1.73155 | + | NA | 10 | -6400.02 | 12820 | 681 | 0.00 |
| 1.24439 | NA | + | NA | NA | 1.72211 | + | NA | 9 | -6406.77 | 12832 | 693 | 0.00 |
| 1.15851 | NA | + | 5.46499 | -3.37034 | 1.76940 | NA | NA | 10 | -6410.93 | 12842 | 703 | 0.00 |
| 3.73133 | NA | NA | 1.61453 | -1.16842 | 1.05165 | + | -0.01247 | 7 | -6429.07 | 12872 | 734 | 0.00 |
| 3.82510 | NA | NA | 0.58499 | NA | 1.03824 | + | -0.01275 | 6 | -6433.41 | 12879 | 740 | 0.00 |
| 3.92611 | NA | NA | NA | NA | 1.01243 | + | -0.01306 | 5 | -6436.49 | 12883 | 744 | 0.00 |
| 3.91955 | NA | NA | NA | 0.09117 | 1.01492 | + | -0.01304 | 6 | -6436.41 | 12885 | 746 | 0.00 |
| 3.94845 | + | NA | 1.50861 | -1.13237 | 1.05225 | NA | -0.01186 | 7 | -6442.52 | 12899 | 760 | 0.00 |
| 4.03995 | + | NA | 0.51053 | NA | 1.03918 | NA | -0.01213 | 6 | -6446.56 | 12905 | 767 | 0.00 |
| 4.12828 | + | NA | NA | NA | 1.01651 | NA | -0.01240 | 5 | -6448.89 | 12908 | 769 | 0.00 |
| 4.12510 | + | NA | NA | 0.04435 | 1.01774 | NA | -0.01238 | 6 | -6448.87 | 12910 | 771 | 0.00 |
| 1.26556 | NA | + | 2.41657 | NA | 1.77916 | NA | NA | 9 | -6446.29 | 12911 | 772 | 0.00 |
| 4.66609 | NA | + | 1.84768 | -1.44471 | NA | NA | -0.01634 | 10 | -6476.11 | 12972 | 834 | 0.00 |
| 4.75354 | NA | + | 0.51714 | NA | NA | NA | -0.01660 | 9 | -6482.26 | 12983 | 844 | 0.00 |
| 4.79777 | NA | + | NA | NA | NA | NA | -0.01675 | 8 | -6484.39 | 12985 | 846 | 0.00 |
| 4.79863 | NA | + | NA | -0.04698 | NA | NA | -0.01676 | 9 | -6484.36 | 12987 | 848 | 0.00 |
| 1.37147 | NA | + | NA | 0.79089 | 1.76495 | NA | NA | 9 | -6488.22 | 12994 | 856 | 0.00 |
| 1.38032 | NA | + | NA | NA | 1.75572 | NA | NA | 8 | -6494.25 | 13005 | 866 | 0.00 |
| 3.87169 | NA | NA | 1.66306 | -1.23005 | 1.09036 | NA | -0.01242 | 6 | -6523.44 | 13059 | 920 | 0.00 |
| 3.97077 | NA | NA | 0.57920 | NA | 1.07633 | NA | -0.01271 | 5 | -6527.99 | 13066 | 927 | 0.00 |
| 4.07073 | NA | NA | NA | NA | 1.05077 | NA | -0.01302 | 4 | -6530.84 | 13070 | 931 | 0.00 |
| 4.06591 | NA | NA | NA | 0.06734 | 1.05262 | NA | -0.01300 | 5 | -6530.80 | 13072 | 933 | 0.00 |
| 5.07439 | + | NA | NA | -0.37439 | NA | + | -0.01719 | 6 | -6534.12 | 13080 | 942 | 0.00 |
| 5.05848 | + | NA | NA | NA | NA | + | -0.01715 | 5 | -6535.48 | 13081 | 942 | 0.00 |
| 5.09362 | + | NA | -0.28021 | NA | NA | + | -0.01724 | 6 | -6534.79 | 13082 | 943 | 0.00 |
| 5.06070 | + | NA | 0.14902 | -0.49216 | NA | + | -0.01715 | 7 | -6534.06 | 13082 | 944 | 0.00 |
| 5.03113 | NA | NA | NA | -0.36239 | NA | + | -0.01789 | 5 | -6598.70 | 13207 | 1069 | 0.00 |
| 5.01576 | NA | NA | NA | NA | NA | + | -0.01785 | 4 | -6599.92 | 13208 | 1069 | 0.00 |
| 5.04574 | NA | NA | -0.23949 | NA | NA | + | -0.01793 | 5 | -6599.44 | 13209 | 1070 | 0.00 |
| 5.00856 | NA | NA | 0.24619 | -0.55696 | NA | + | -0.01783 | 6 | -6598.54 | 13209 | 1070 | 0.00 |
| 5.25348 | + | NA | NA | -0.41364 | NA | NA | -0.01720 | 5 | -6610.87 | 13232 | 1093 | 0.00 |
| 1.65442 | + | NA | 4.06997 | -2.63980 | 1.67902 | + | NA | 7 | -6609.23 | 13232 | 1094 | 0.00 |
| 5.23626 | + | NA | NA | NA | NA | NA | -0.01716 | 4 | -6612.45 | 13233 | 1094 | 0.00 |
| 5.27614 | + | NA | -0.31998 | NA | NA | NA | -0.01727 | 5 | -6611.60 | 13233 | 1095 | 0.00 |
| 5.24144 | + | NA | 0.13126 | -0.51737 | NA | NA | -0.01717 | 6 | -6610.83 | 13234 | 1095 | 0.00 |
| 1.75808 | + | NA | 1.80855 | NA | 1.68257 | + | NA | 6 | -6629.65 | 13271 | 1133 | 0.00 |
| 1.89312 | + | NA | NA | 0.59448 | 1.66205 | + | NA | 6 | -6653.94 | 13320 | 1181 | 0.00 |
| 1.91356 | + | NA | NA | NA | 1.65289 | + | NA | 5 | -6657.11 | 13324 | 1186 | 0.00 |
| 1.80605 | + | NA | 4.06404 | -2.66321 | 1.70010 | NA | NA | 6 | -6669.73 | 13351 | 1213 | 0.00 |
| 1.45409 | NA | NA | 4.34256 | -2.80380 | 1.74342 | + | NA | 6 | -6682.02 | 13376 | 1237 | 0.00 |
| 1.91109 | + | NA | 1.78249 | NA | 1.70375 | NA | NA | 5 | -6689.78 | 13390 | 1251 | 0.00 |
| 5.23149 | NA | NA | NA | -0.40582 | NA | NA | -0.01804 | 4 | -6696.08 | 13400 | 1262 | 0.00 |
| 5.21461 | NA | NA | NA | NA | NA | NA | -0.01800 | 3 | -6697.52 | 13401 | 1262 | 0.00 |
| 5.24922 | NA | NA | -0.27800 | NA | NA | NA | -0.01809 | 4 | -6696.91 | 13402 | 1263 | 0.00 |
| 5.20910 | NA | NA | 0.24423 | -0.59883 | NA | NA | -0.01799 | 5 | -6695.93 | 13402 | 1263 | 0.00 |
| 1.56066 | NA | NA | 1.94306 | NA | 1.74838 | + | NA | 5 | -6704.09 | 13418 | 1280 | 0.00 |
| 2.04425 | + | NA | NA | 0.56638 | 1.68313 | NA | NA | 5 | -6712.75 | 13436 | 1297 | 0.00 |
| 2.06336 | + | NA | NA | NA | 1.67435 | NA | NA | 4 | -6715.53 | 13439 | 1300 | 0.00 |
| 1.70074 | NA | NA | NA | 0.64940 | 1.72808 | + | NA | 5 | -6730.83 | 13472 | 1333 | 0.00 |
| 1.72229 | NA | NA | NA | NA | 1.71834 | + | NA | 4 | -6734.45 | 13477 | 1338 | 0.00 |
| 1.60226 | NA | NA | 4.37919 | -2.85790 | 1.77880 | NA | NA | 5 | -6761.63 | 13533 | 1395 | 0.00 |
| 1.71169 | NA | NA | 1.93333 | NA | 1.78404 | NA | NA | 4 | -6783.51 | 13575 | 1436 | 1.21571266508283e-312 |
| 1.85177 | NA | NA | NA | 0.62428 | 1.76351 | NA | NA | 4 | -6808.99 | 13626 | 1487 | 9.88131291682493e-324 |
| 1.87223 | NA | NA | NA | NA | 1.75408 | NA | NA | 3 | -6812.19 | 13630 | 1492 | 0.00 |
| 2.28536 | + | + | 5.33138 | -3.64906 | NA | + | NA | 11 | -6807.54 | 13637 | 1499 | 0.00 |
| 2.40730 | + | + | 2.02842 | NA | NA | + | NA | 10 | -6840.32 | 13701 | 1562 | 0.00 |
| 2.49025 | + | + | NA | 0.41161 | NA | + | NA | 10 | -6865.96 | 13752 | 1613 | 0.00 |
| 2.49206 | + | + | NA | NA | NA | + | NA | 9 | -6867.27 | 13753 | 1614 | 0.00 |
| 2.46645 | + | + | 5.34448 | -3.68524 | NA | NA | NA | 10 | -6869.10 | 13758 | 1620 | 0.00 |
| 2.59057 | + | + | 2.00867 | NA | NA | NA | NA | 9 | -6901.33 | 13821 | 1682 | 0.00 |
| 1.96409 | NA | + | 5.32749 | -3.68502 | NA | + | NA | 10 | -6911.45 | 13843 | 1704 | 0.00 |
| 2.67227 | + | + | NA | 0.38535 | NA | NA | NA | 9 | -6925.72 | 13869 | 1731 | 0.00 |
| 2.67374 | + | + | NA | NA | NA | NA | NA | 8 | -6926.83 | 13870 | 1731 | 0.00 |
| 2.08625 | NA | + | 1.99187 | NA | NA | + | NA | 9 | -6942.88 | 13904 | 1765 | 0.00 |
| 2.16896 | NA | + | NA | 0.37271 | NA | + | NA | 9 | -6966.34 | 13951 | 1812 | 0.00 |
| 2.17095 | NA | + | NA | NA | NA | + | NA | 8 | -6967.35 | 13951 | 1812 | 0.00 |
| 2.14035 | NA | + | 5.34307 | -3.73481 | NA | NA | NA | 9 | -6996.49 | 14011 | 1872 | 0.00 |
| 2.26505 | NA | + | 1.96223 | NA | NA | NA | NA | 8 | -7027.19 | 14070 | 1932 | 0.00 |
| 2.34774 | NA | + | NA | NA | NA | NA | NA | 7 | -7049.79 | 14114 | 1975 | 0.00 |
| 2.34615 | NA | + | NA | 0.33471 | NA | NA | NA | 8 | -7049.02 | 14114 | 1975 | 0.00 |
| 2.37013 | + | NA | 3.55903 | -2.79290 | NA | + | NA | 6 | -7094.58 | 14201 | 2063 | 0.00 |
| 2.48142 | + | NA | 1.16501 | NA | NA | + | NA | 5 | -7111.72 | 14233 | 2095 | 0.00 |
| 2.57394 | + | NA | NA | NA | NA | + | NA | 4 | -7120.37 | 14249 | 2110 | 0.00 |
| 2.57280 | + | NA | NA | 0.04029 | NA | + | NA | 5 | -7120.36 | 14251 | 2112 | 0.00 |
| 2.54943 | + | NA | 3.54525 | -2.82119 | NA | NA | NA | 5 | -7151.03 | 14312 | 2173 | 0.00 |
| 2.66240 | + | NA | 1.12687 | NA | NA | NA | NA | 4 | -7167.95 | 14344 | 2205 | 0.00 |
| 2.75104 | + | NA | NA | NA | NA | NA | NA | 3 | -7175.78 | 14358 | 2219 | 0.00 |
| 2.75101 | + | NA | NA | 0.00111 | NA | NA | NA | 4 | -7175.78 | 14360 | 2221 | 0.00 |
| 2.14458 | NA | NA | 3.88912 | -3.01454 | NA | + | NA | 5 | -7185.84 | 14382 | 2243 | 0.00 |
| 2.26129 | NA | NA | 1.30733 | NA | NA | + | NA | 4 | -7204.76 | 14418 | 2279 | 0.00 |
| 2.36251 | NA | NA | NA | NA | NA | + | NA | 3 | -7215.08 | 14436 | 2298 | 0.00 |
| 2.36023 | NA | NA | NA | 0.08258 | NA | + | NA | 4 | -7215.04 | 14438 | 2299 | 0.00 |
| 2.33103 | NA | NA | 3.92064 | -3.08166 | NA | NA | NA | 4 | -7263.60 | 14535 | 2397 | 0.00 |
| 2.45137 | NA | NA | 1.28122 | NA | NA | NA | NA | 3 | -7282.49 | 14571 | 2432 | 0.00 |
| 2.55009 | NA | NA | NA | NA | NA | NA | NA | 2 | -7291.95 | 14588 | 2449 | 0.00 |
| 2.54900 | NA | NA | NA | 0.04044 | NA | NA | NA | 3 | -7291.94 | 14590 | 2451 | 0.00 |
